# Supplementary material for: Causal effects of blood lipids on amyotrophic lateral sclerosis: a Mendelian randomization study
Source: Hum Mol Genet. 2018 Nov 16;28(4):688–97. doi: 10.1093/hmg/ddy384 (PMC6360326; doi:10.1093/hmg/ddy384)
Supplement: Supplementary Data [file ddy384_supp.zip › 20180906_Supplementary_Material(HMG).doc]

**Supplementary Materials for “Causal Effects of Blood Lipids on Amyotrophic Lateral Sclerosis: A Mendelian Randomization Study”**

Ping Zeng^1*^, Xiang Zhou^2,3*^

**^1^**Department of Epidemiology and Biostatistics, Xuzhou Medical University, Xuzhou, 221004, Jiangsu, China

**^2^**Department of Biostatistics, University of Michigan, Ann Arbor, 48109, Michigan, USA

**^3^**Center for Statistical Genetics, University of Michigan, Ann Arbor, Michigan, USA

*To whom correspondence should be addressed at:

Department of Epidemiology and Biostatistics, Xuzhou Medical University, Xuzhou, 221004, Jiangsu, China. Tel: 86-516-83262659, Email: zpstat@xzhmu.edu.cn (P.Z.)

Department of Biostatistics, University of Michigan, Ann Arbor, 48109, Michigan, USA. Tel: 734-764-5722, Email: [xzhousph@umich.edu](mailto:xzhousph@umich.edu) (X.Z.)

Detailed Methods

Effect sizes of lipids on LAS are comparable to some other risk factors

The Supplementary Table S1 provides a comprehensive summary of the effect sizes of various risk factors on ALS that have been examined in previous studies. Those factors include physical activity, body mass index (BMI), smoking, alcohol drinking, metals and pesticides exposure as well as lipids — high density lipoprotein (HDL), low density lipoprotein (LDL), total cholesterol (TC) and triglycerides (TG). Because different studies vary in their study designs, definition of exposure of interest, controlling of covariates, and because some risk factors are binary (e.g. smoking) and some are continuous (e.g. lipid levels) but are measured on different units (e.g. some measured in the unit of mmol/l and some in *μ*g/l), it is challenging to compare directly the effect sizes of lipids on ALS with the effect sizes of other factors across various studies. We did, however, found one particular ALS observational study that is relatively large in sample size (~2,400 individuals), that provides the effect size estimates for both lipids and some other risk factor, and that also provides standard deviations of these risk factors . The availability of standard deviation of these risk factors in that study allow us to scale the effect size estimates with respect to one standard deviation change of the risk factor, which would then allow us to compare effect size across different factors. In that study, the effect sizes of TC and LDL were estimated to be 0.22 and 0.37, respectively; while the effect size of body mass index (BMI), an important risk factor for ALS and for various other diseases , was estimated to be 0.38. Therefore, the effect sizes of lipids on LAS are comparable to some other important risk factors.

Mendelian randomization Assumptions

Mendelian randomization is an important statistical method to investigate the causal relationship between an exposure variable (i.e. lipid level) and an outcome variable (i.e. ALS) in observational studies. Mendelian randomization selects genetic variants as instrumental variables and applies them to produce an unbiased estimate of the causal effect from the exposure variable onto the outcome variable. An important step of Mendelian randomization is to carefully select genetic variants to serve as valid instruments. Each of these selected genetic variants has to satisfy three conditions (or assumptions) (Fig. S1) : (**i**) **relevance**: it is strongly associated with the exposure of interest; (**ii**) **independence**: it is not associated with any other confounders that may be associated with both the exposure and outcome; (**iii**) **exclusion restriction**: it only influences the outcome by the path of the exposure and does not have horizontal pleiotropic or directional effects. Note that, the first condition (i.e. the relevance assumption) can be directly tested based on the observed data while the second two conditions (i.e. the independence and exclusion restriction assumptions) are hard to validate due to variables that may not be recorded in the study — in the present paper we will instead validate the last two conditions through various sensitivity analyses. In the past decade large scale genome-wide association studies (GWASs) have identified many single nucleotide polymorphisms (SNPs) that are strongly and robustly associated with lipids , making it feasible to choose appropriate genetic variants that can serve as valid instruments .

Select instruments for HDL, LDL, TC and TG in the European population

We selected SNPs to serve as instrumental variables for four lipid traits in the European population. The four lipid traits include HDL, LDL, TC, and TG. For SNP selection, we first obtained GWAS association summary statistics for the four lipid traits from the public portal (http://csg.sph.umich.edu/abecasis/public/lipids2013/) of the Global Lipids Genetics Consortium (GLGC) study . The GLGC study is the largest GWAS performed to date on lipid traits. It is a meta-analysis that analyzed ~2.5 million genotyped and imputed SNPs on 188,577 individuals of European ancestry collected from 63 sub-studies and from 23 sub-studies from previous lipid GWAS . The GLGC study properly adjusted for sex effect, non-linear effect of age (by incorporating age and age^2^ as covariates) as well as population stratification . Within the GLGC study, we carefully selected a small set of index SNPs that are independent with each other and that are associated with either HDL, LDL, TC or TG at the genome-wide significance level (*p* < 5.00E-8). We do so by applying the clumping procedure of PLINK (version v1.90b3.38) , where, following , we set both the significance threshold for index SNPs and the secondary significance threshold for clumped SNPs to be 5.00E-8, and set the linkage disequilibrium (LD) threshold and the physical distance threshold for clumping to be 0.001 and 10,000, respectively. The LD are estimated using a reference panel of 503 individuals of European (EUR) ancestry in the 1000 Genomes Project .

After clumping, we obtained 88, 78, 86 and 54 independent index association SNPs (with *p* < 5.00E-8) as instrumental variables for HDL (Table S2), LDL (Table S3), TC (Table S4) and TG (Table S5), respectively. For these instruments, we obtained from the GLGC study the association summary statistics for the four lipids in terms of effect allele, effect size, standard error, p value, and sample size. To estimate the causal effect of each of the four lipids on ALS, we also extracted association summary statistics of these index SNPs for ALS from an ALS GWAS, which is also performed in the European population . The ALS GWAS is a case-control study that analyzed ~8.7 million genotyped and imputed SNPs on 41,689 individuals (14,791 cases and 26,898 controls) of European ancestry collected from 49 sub-studies (Table S14). We obtained summary statistics from the ALS GWAS in the form of effect allele, effect size (in terms of log OR), and standard error (Tables S2-5). The ALS GWAS contains summary statistics for all the index SNPs selected from GLGC .

Select instruments for HDL, LDL, TC and TG in the East Asian population

We also selected SNPs to serve as instruments for HDL, LDL, TC and TG in the East Asian population. To do so, we extracted association summary statistic results from an East Asian lipid GWAS performed on Japanese individuals . The East Asian lipid GWAS analyzed a total of 5,961,600 genotyped and imputed SNPs, on 70,657 individuals for HDL, 72,866 individuals for LDL, 128,305 individuals for TC and 105,597 individuals for TG (Table S15). The study properly controlled for the effects of sex, age, genetic ancestry, disease status and other trait-specific covariates whenever available.

We obtained from the East Asian lipid GWAS a set of 31, 22, 32 and 26 index association SNPs to serve as instrumental variables for HDL (Table S6), LDL (Table S7), TC (Table S8) and TG (Table S9), respectively. For these index SNPs, we extracted the corresponding association summary statistics from an East Asian ALS GWAS performed on Chinese individuals . The East Asian ALS GWAS analyzed 6,613,544 genotyped and imputed SNPs on up to 4,084 individuals (1,234 cases and 2,850 controls). With the only exception of three SNPs (i.e. rs189213544 for HDL, rs147943615 for LDL and rs78295286 for TC), all other index SNPs for the four lipid traits from the East Asian lipid GWAS have association results in the East Asian ALS GWAS . For the three index SNPs with missing ALS association statistics (i.e. rs189213544, rs147943615 and rs78295286), we have attempted to replace them with proxy variants that are in high LD with them. Specifically, for each of the three target SNPs, we extracted all SNPs within 1 Mb distance of the target SNP and identified among them a proxy SNP that is most correlated with the target SNP based on LD information obtained from 504 individuals with Asian ancestry in the 1000 Genomes Project . Unfortunately, none of the proxy SNPs identified were highly correlated with the target SNPs: the correlation coefficient between the proxy SNP and the target SNP is only 0.151 for rs138761799 and rs189213544, 0.408 for rs149726725 and rs147943615, and 0.481 for rs79124660 and rs78295286, respectively. Therefore, to minimize the potential bias due to low quality of these proxy SNPs, we decided to focus only on index SNPs that have no missing information in the East Asian ALS GWAS in the following Mendelian randomization analysis. In total, we analyzed 30, 21, 31 and 26 instrumental variables for HDL, LDL, TC and TG, respectively.

Unify lipid level measurement unit in the two populations

In each population, for each lipid trait in turn, we estimate the causal effect of the lipid on ALS in terms of the odds ratio for one-unit change of lipid level. We define the unit of lipid as the standard deviation (SD) of the examined lipid trait. Because the SD of each lipid trait differs slightly between the European and East Asian populations, we choose to use the average SDs between the two populations to serve as the final unit. Specifically, the SDs of the four lipids in the European population are estimated to be 14.7 mg/dL, 37.0 mg/dL, 42.6 mg/dL and 86.8 mg/dL for HDL, LDL, TC and TG, respectively, based on the GLGC study (Table S13) . The SDs of the four lipids in the East Asian population are estimated to be 15.4 mg/dL, 41.0 mg/dL, 38.6 mg/dL and 71.9 mg/dL for HDL, LDL, TC and TG, respectively, based on the East Asian lipids GWAS (Table S15) .

To make the causal effect estimates comparable between the two populations, we obtained the average SDs for the four lipids and used them as the final units (i.e. 15.1 mg/dL for HDL, 39.0 mg/dL for LDL, 40.6 mg/dL for TC and 79.3 mg/dL for TG). Afterwards, we converted the original lipid GWAS association results that were measured based on SD of lipid levels within the given population to the new association results that were measured based on the average SD of lipid levels between the two populations, by simply multiplying the marginal effect size and standard error of the index SNPs by the ratio of the averaged lipid SD and the original lipid SD within the population. For example, we recomputed the effect size and standard error of each LDL index SNP in the GLGC study or in the East Asian lipid study by multiplying a factor of 0.95 (= 37.0/39.0) or 1.05 (= 41.0/39.0), respectively.

Alternatively, instead of mg/dL as used for the unit of lipids in the present, one can employed another commonly-used unit (i.e. mmol/L) for lipids. There exists a simple relationship between mg/dL and mmol/L (http://www.endmemo.com/medical/unitconvert/): mg/dL=mmol/L×38.61. In our paper, to avoid complication and confusion, we only focused on the unit of mmol/L and estimated the causal effects of lipids. Indeed, the estimates of the causal effects of lipids with the unit of mmol/L can be easily transformed into the estimates of the causal effects of lipids with the unit of mmol/L by utilizing the relationship above.

Note that the scale transformation above does not influence the p value of the estimated causal effect, as the transformation changes the causal effect estimates and the standard errors at the same scale. We report results based on the transformed summary statistics (with the unit of mg/dL) throughout the paper.

Estimate causal effect using the inverse-variance weighted Mendelian randomization methods

Here, we describe in detail the two-sample Mendelian randomization analysis, which is a special form of the instrumental variable analysis. The two-sample Mendelian randomization method developed from the causal inference field allows the exposure variable (e.g. LDL) and the outcome variable (i.e. ALS) to be measured on two separate data sets using only summary statistics. Specifically, we first matched the risk allele between the lipid GWAS and the ALS GWAS to ensure that the SNP effects on either the lipid or ALS are measured with respect to the same allele. For each lipid in turn, we examined the set of SNPs that were selected as instrumental variables and removed among them those that were associated with ALS with a marginal p value below 0.05 after Bonferroni correction (i.e. p < 0.05/*k*, where *k* is the number of the instrumental variables for the given lipid). With the remaining instruments, we applied both the fixed-effects and random-effects versions of the inverse variance weighted (IVW) methods to pool results from individual SNPs to estimate the overall causal effect of each lipid on ALS.

For exposure **x**, let’s assume that the effect size estimate and its variance for instrumental variable *i* are $\hat{\beta}_{i}^{x}$ and $\text{Var}(\hat{\beta}_{i}^{x})$ (*i* = 1, 2, …, *k*), respectively. We denote ALS disease status as **y** and denote the effect size estimate and its variance for the same instrumental variable *i* on y as $\hat{\beta}_{i}^{y}$ and $\text{Var}(\hat{\beta}_{i}^{y})$, respectively. Let the causal effect of **x** (e.g. LDL) on **y** (i.e. ALS) be *θ*. Following tradition, in what follows, we define *θ* as the effect of per unit increase of lipid on the log odds ratio (OR) of ALS. We first estimated *θ* based on a single instrumental variable *i* as

$\hat{\theta}_{i} = \hat{\beta}_{i}^{y}/\hat{\beta}_{i}^{x}$.

The asymptotic variance of $\hat{\theta}_{i}$ can be estimated via the Delta method . We next combined the evidence from all instrumental variables using the IVW methods to obtain a final estimate of *θ* . Two versions of IVW methods exist: a fixed-effects version and a random-effects version . In both the fixed-effects and random-effects IVW methods we estimate the causal effect *θ* by pooling all SNPs as

$\hat{\theta}_{\text{ivw}} = \frac{\sum_{i} \text{Var}(\hat{\beta}_{i}^{y})^{-1}\hat{\beta}_{i}^{y}\hat{\beta}_{i}^{x}}{\sum_{i} \text{Var}(\hat{\beta}_{i}^{y})^{-1}(\hat{\beta}_{i}^{x})^{2}}$.

In effect, the IVW estimate $\hat{\theta}_{\text{ivw}}$ is obtained by treating $\hat{\beta}_{i}^{y}$ as the response and $\hat{\beta}_{i}^{x}$ as the exposure by fitting a regression model with heterogeneous variance weights that are proportional to $\text{Var}(\hat{\beta}_{i}^{y})$. Note that we do not impose an intercept in the above regression model in order to satisfy the assumption that the response is zero whenever the exposure is zero. The fixed-effects version of IVW assumes that the causal effect estimate produced by using any SNP instrument is the same and that there is no effect heterogeneity across SNP instruments . In contrast, the random-effects version of IVW allows for effect heterogeneity across SNP instruments by introducing an extra over-dispersion parameter in the Mendelian randomization model . As a result, compared to the fixed-effects IVW, the random-effects IVW yields the same point estimate but with a slightly larger standard error. To directly measure effect size heterogeneity across SNP instruments, we also employed both Q and I^2^ statistics in our main analysis .

Sensitivity analyses

For our main Mendelian randomization analysis on the causal effect of LDL on ALS, we also performed several sensitivity analyses to examine results robustness and guard against various modeling misspecifications. (**i**) To guard against the possibility that some associated instruments for one lipid may exhibit their causal effects on ALS through the other lipids, we performed Mendelian randomization for each lipid using lipid-specific instruments. In particular, for each lipid in turn, we obtained a set of lipid-specific instruments by removing these also employed for the other three lipids. We then performed Mendelian randomization analysis using these lipid-specific instruments. (**ii**) We conducted a leave-one-out (LOO) cross validation analysis to examine whether there are instrumental outliers and whether these outliers affect results . In the LOO analysis, we removed one instrumental variable at a time and computed the causal effect estimate with the remaining instruments using the IVW methods. The discrepancy between the LOO causal effect estimates obtained by leaving one instrument out and the original causal effect estimate with all instruments represents the influence of the instrument that was left out. (**iii**) In addition to the IVW methods, we also perform the median-based method to estimate the causal effects of LDL on ALS. Compared with the IVW methods, the median-based method is more robust against outlier instruments and can offer a consistent estimate for the causal effect even when as many as 50% instrumental variables are invalid . (**iv**) We carry out MR-Egger regression to examine the assumption of directional pleiotropic effects . Specifically, the MR-Egger regression is a modification of the IVW methods and assumes that

$\hat{\beta}_{i}^{y} =\alpha+ \hat{\beta}_{i}^{x}\theta_{\text{Egger}} + e_{i}, e_{i} \sim N(0, \text{τVar}(\hat{\beta}_{i}^{y}))$.

where *α* is the intercept term, $\theta_{\text{Egger}}$ is the MR-Egger version of the pooled causal effect $\theta$, and $\text{τ}$ is the residual variance. Compared with the IVW methods, the MR-Egger regression introduces an extra intercept term *α*, which, under the InSIDE (Instrument Strength Independent of Direction Effect, InSIDE) assumption, can be interpreted as the average pleiotropic effect of the instrumental variables . In the presence of balanced pleiotropy, *α* will equal to zero, and the MR-Egger regression reduces to the IVW method with the MR-Egger estimate $\hat{\theta}_{\text{Egger}}$ being a consistent estimate of $\theta$. In contrast, deviation of *α* from zero would suggest either directional pleiotropy or invalid InSIDE assumption. Therefore, in the MR-Egger regression, an intercept term that is estimated to be statistically significantly from zero would suggest potential violation of IVW method assumption and subsequently biased causal effect estimates from the IVW methods.

Multivariable Mendelian randomization analysis

In our main analyses we have identified LDL to be causally associated with ALS. We note that some previous observational studies have shown that the ratio of LDL/HDL, in addition to LDL alone, is also correlated with ALS status . While it is tempting to examine the causal effect of the ratio of LDL/HDL on ALS in the present study, we acknowledge that it would be challenging to do so because there is currently no GWAS summary statistics available for the ratio of LDL/HDL. However, instead of directly investigating the causal effect of LDL/HDL on ALS, we can indirectly examine this relationship by estimating the causal effect of LDL on ALS while controlling for HDL. Intuitively, if LDL/HDL is high for an individual, then the residual value of LDL after controlling for HDL would also be high for the individual. Therefore, if LDL/HDL is associated with ALS status, then LDL after controlling for HDL will also likely be associated with ALS. To test the causal effect of LDL on ALS after controlling for HDL, we performed an additional analysis which is in a similar spirit to the multivariable Mendelian randomization done before . Here, we first regressed out the HDL effects from LDL through a linear regression model and treated the resulting LDL residuals as the new exposure variable. We then performed Mendelian randomization analysis using ALS and the LDL residual variable with a linear regression model and obtained an estimate of the causal effect for LDL residuals. In both these regression models we set the regression weight as the inverse variance of the variant effect size of ALS following .

Finally, for LDL, we also performed a residual-based (or joint) multivariable Mendelian randomization analysis by treating HDL and TG as covariates. Note that we cannot include TC in addition to HDL and TG as covariate in the model because LDL level is completely determined by the other three lipids and is highly correlated with TC (correlation coefficient *r* = 0.992 and 0.787 in the European and East Asian populations). The multivariate Mendelian randomization analysis tests for the causal effects of LDL on ALS while controlling for the other lipid effects, thus providing a way to control for instrumental pleiotropic effects on other lipids.

Exclude reverse causality between LDL and ALS

We carried out a reverse causal inference to formally exclude the possibility that the inferred causal relationship between LDL and ALS was due to a causal effect of ALS on LDL. To do so, we performed a Mendelian randomization analysis in the reverse direction by using ALS associated SNPs as instrumental variables. In the European population, with the same clumping procedure described above, we obtained a total of 5 ALS associated SNPs (*p* < 5.00E-8) to serve as instruments . For these SNP instruments, we extracted their association summary statistics with LDL from GLGC . For 4 SNP instruments that do not have corresponding association information with LDL, we replaced them with proxy SNPs following the same strategy described above. Afterwards, we applied both the fixed-effects and mixed-effects IVW methods to estimate the causal effect of ALS on LDL. In the East Asian population, no SNP is associated with ALS at a significance level below 5.00E-8 . Therefore, we chose the top associated SNP (rs12464221; *p* value = 1.13E-6) as a single instrument for ALS and applied Equation (1) directly to estimate the causal effect of ALS on LDL.

Select instruments for HDL, LDL, TC and TG in the Chinese population

In the main analyses for the East Asian population, we have paired a Japanese lipids GWAS with a Chinese ALS GWAS. To alleviate the concern on the potential genetic difference between the Japanese and Chinese groups, we also performed analyses using a two-stage lipids GWAS performed on Chinese individuals . The discovery stage of this Chinese lipid GWAS analyzed four sub-studies with up to 8,344 Chinese individuals. The four sub-studies include the China Atherosclerosis Study (CAS), the Beijing Atherosclerosis Study (BAS), Genetic Epidemiology Network of Salt-Sensitivity (GenSalt) study, and the Guangxi Fangchenggang Area Male Health and Examination Survey (FAMHES). The replication stage of this Chinese lipid GWAS further analyzed 14,739 individuals from the China Cardiovascular Health Study (CCHS). In total, the Chinese lipid GWAS analyzed ~2.5 million genotyped or imputed SNPs. The study properly controlled for sex effects, non-linear effects of age (by incorporating age and age^2^ as covariates), effects of body mass index, as well as population stratification .

Following the same procedure described in the previous section, we selected from the Chinese lipid GWAS a set of 6, 7, 14 and 9 index association SNPs (Tables S11-12) to serve as instrumental variables for HDL, LDL, TC and TG, respectively. For these instruments, we obtained from the Chinese lipid GWAS the association summary statistics in the form of effect allele, effect size, standard error, p value, and sample size (Table S12). We also extracted association summary statistics of these SNPs with ALS from the same Chinese ALS GWAS we used in the main analyses . With the only exception of two SNPs (rs9357121 and rs11066280), all index SNPs for the four lipid traits from the Chinese lipid GWAS have association results in the Chinese ALS GWAS . For these two SNPs without corresponding ALS association statistics, we replaced these SNPs with two proxy variants that are in high LD with them. Specifically, for either rs9357121 or rs11066280, we extracted SNPs that are located within 1 Mb distance of the target SNP. Among these extracted SNPs, we further identified among them a proxy SNP that is most correlated with the target SNP using LD information obtained from 504 individuals with Asian ancestry in the 1000 Genomes Project . This way, we replaced rs9357121 with a proxy SNP rs115034963 (correlation coefficient *r* = 0.96) and replaced rs11066280 with a proxy SNP rs11066150 (*r* = 0.62) (Table S12). We used the two proxy SNPs together with the remaining index SNPs for the following Mendelian randomization analysis (Fig. S4).

**Table S1. Summary of disease risk factors and their effect sizes on ALS estimated in previous studies**

| Factors | OR | 95% Confidence Interval | |  | | Reference |
| --- | --- | --- | --- | --- | --- | --- |
|  |  | Lower | Upper |  |  |  |
| **Use cholesterol lowering agents** | 0.60 | 0.40 | 0.90 |  |  |  |
| **Overweight** | 0.70 | 0.50 | 1.00 |  |  |  |
| **BMI (kg/m^2^; sd = 3.56)** | 0.90 | 0.90 | 1.00 |  |  |  |
| **TC women (mmol/l; sd = 1.00)** | **0.80** | NA | NA |  |  |  |
| **LDL women (mmol/l; sd = 0.90)** | **0.50** | NA | NA |  |  |  |
| **HDL women (mmol/l; sd = 0.41)** | **2.50** | NA | NA |  |  |  |
| **LDL/HDL women (sd = 0.99)** | 0.40 | 0.30 | 0.60 |  |  |  |
| **Homocysteine women (μg/l)** | 0.90 | 0.90 | 1.00 |  |  |  |
| High-sensitivity C-reactive protein women (mg/l) | 1.00 | NA | NA |  |  |  |
| **TC men (mmol/l; sd = 1.10)** | 0.60 | NA | NA |  |  |  |
| **LDL men (mmol/l; sd = 0.93)** | 0.70 | NA | NA |  |  |  |
| **HDL men (mmol/l; sd = 0.33)** | 2.10 | NA | NA |  |  |  |
| **LDL/HDL men (sd = 1.00)** | 0.50 | 0.40 | 0.60 |  |  |  |
| **Homocysteine men (μg/l)** | 0.90 | 0.80 | 0.90 |  |  |  |
| High-sensitivity C-reactive protein men (mg/l) | 1.00 | NA | NA |  |  |  |
| BMI men (kg/m^2^) | 0.93 | 0.86 | 0.99 |  |  |  |
| BMI women (kg/m^2^) | 0.99 | 0.95 | 1.03 |  |  |  |
| **Waist/hip ratio women** | 0.48 | 0.25 | 0.93 |  |  |  |
| **BMI** | 0.21 | 0.14 | 0.27 |  |  |  |
| **Overweight** | 0.75 | 0.65 | 0.85 |  |  |  |
| **Slimness** | 2.21 | 1.40 | 3.47 |  |  |  |
| **Varsity athletics** | 1.70 | 1.04 | 2.76 |  |  |  |
| **Overweight** | 2.22 | 1.18 | 4.17 |  |  |  |
| **Normal/underweight** | 2.48 | 1.36 | 4.53 |  |  |  |
| **Leisure time physical activity** | 1.08 | 1.02 | 1.14 |  |  |  |
| **Current smokers** | 1.38 | 1.02 | 1.88 |  |  |  |
| **Current alcohol consumption** | 0.52 | 0.40 | 0.75 |  |  |  |
| **Ever smokers** | 2.00 | 1.30 | 3.20 |  |  |  |
| **Current smokers** | 3.50 | 1.90 | 6.40 |  |  |  |
| Former smokers | 1.50 | 0.90 | 2.40 |  |  |  |
| Former smokers | 1.00 | 0.70 | 1.40 |  |  |  |
| **Current smoker**s | 1.70 | 1.10 | 2.60 |  |  |  |
| Ever smokers | 1.04 | 0.80 | 1.34 |  |  |  |
| **Ever smokers women** | 1.53 | 1.04 | 2.23 |  |  |  |
| **Current smokers women** | 1.67 | 1.24 | 2.24 |  |  |  |
| **Current smokers men** | 0.69 | 0.49 | 0.99 |  |  |  |
| **Former smokers** | 1.44 | 1.23 | 1.68 |  |  |  |
| **Current smokers** | 1.42 | 1.07 | 1.88 |  |  |  |
| **Deployed personnel** | 1.92 | 1.29 | 2.84 |  |  |  |
| **Deployed active duty military** | 2.15 | 1.38 | 3.36 |  |  |  |
| **Deployed Air Force** | 2.68 | 1.24 | 5.78 |  |  |  |
| **Deployed Army** | 2.04 | 1.10 | 3.77 |  |  |  |
| **Deployed Reserves and National Guard** | 2.50 | 0.88 | 7.07 |  |  |  |
| Deployed Navy | 1.48 | 0.62 | 3.57 |  |  |  |
| **Deployed Marine Corps** | 1.13 | 27.00 | 7.79 |  |  |  |
| **Lead exposure** | 1.90 | 1.10 | 3.30 |  |  |  |
| **Premorbid intake of total fat** | 1.14 | 1.07 | 1.23 |  |  |  |
| **Saturated fat** | 1.43 | 1.25 | 1.64 |  |  |  |
| **Trans-fatty acids** | 1.03 | 1.01 | 1.05 |  |  |  |
| **Cholesterol** | 1.08 | 1.05 | 1.12 |  |  |  |
| **Alcohol** | 0.91 | 0.84 | 0.99 |  |  |  |
| **Metals exposure** | 3.65 | 1.15 | 11.60 |  |  |  |
| **Pesticides exposure** | 6.50 | 1.78 | 23.77 |  |  |  |
|  | Case | |  | Control | |  |
|  | Mean | SD |  | Mean | SD |  |
| **HDL (mg/dl)** | 60.00 | 15.00 |  | 60.00 | 20.00 |  |
| **LDL (mg/dl)** | 160.00 | 40.00 |  | 120.00 | 40.00 |  |
| TC (mg/dl) | 250.00 | 50.00 |  | 210.00 | 50.00 |  |
| TG (mg/dl) | 130.00 | 90.00 |  | 130.00 | 80.00 |  |
| **LDL/HDL** | 2.90 | 1.20 |  | 2.10 | 0.90 |  |
| HDL (mg/dl) | 59.30 | 15.50 |  | 59.60 | 16.00 |  |
| LDL (mg/dl) | 128.70 | 38.60 |  | 125.70 | 40.10 |  |
| TC (mg/dl) | 211.10 | 44.10 |  | 208.20 | 48.50 |  |
| TG (mg/dl) | 115.10 | 54.60 |  | 118.10 | 58.90 |  |
| LDL/HDL | 2.17 | 0.89 |  | 2.15 | 1.20 |  |
| HDL (mg/dl) | 46.30 | 12.30 |  | 52.50 | 16.60 |  |
| LDL (mg/dl) | 108.10 | 10.80 |  | 108.70 | 11.30 |  |
| **TG (mg/dl)** | 127.50 | 89.40 |  | 121.30 | 63.80 |  |
| TC (mg/dl) | 202.50 | 45.40 |  | 205.30 | 30.90 |  |
| HDL (mg/dl) | 47.28 | 12.14 |  | 46.21 | 13.37 |  |
| LDL (mg/dl) | 115.56 | 28.72 |  | 120.24 | 26.49 |  |
| **TC (mg/dl)** | 188.17 | 33.93 |  | 197.76 | 30.08 |  |
| TG (mg/dl) | 126.73 | 73.86 |  | 156.08 | 135.08 |  |
| **LDL/HDL** | 2.58 | 0.88 |  | 2.84 | 1.02 |  |
| **Protein (g/dl)** | 7.11 | 0.48 |  | 7.29 | 0.37 |  |
| Glucose (mg/dl) | 98.49 | 24.21 |  | 98.86 | 21.49 |  |

ALS: amyotrophic lateral sclerosis, BMI: body mass index; HDL: high density lipoprotein; LDL: low density lipoprotein; TC: total cholesterol; TG: triglycerides. A more detailed systematic review on the risks of various chemicals and metals on ALS can be found in . An overview of the environmental and genetic epidemiology of ALS can be found in . The SNP effect sizes on ALS can be found from the NHGRI-EBI Catalog of published GWASs at [https://www.ebi.ac.uk](https://www.ebi.ac.uk/). Significant factors are highlighted in bold.

**Table S2.** Index SNPs used as instruments for HDL and the effects, standard errors and association strength for ASL in the European population

| SNP | Chr | Position (GRCh37) | HDL | | | | | |  | ASL | | |
| --- | --- | --- | --- | --- | --- | --- | --- | --- | --- | --- | --- | --- |
|  |  |  | Allele | Beta | SE | *N* | PVE | *F* |  | Allele | Beta | SE |
| rs2250802 | 10 | 113,921,354 | G | 0.0340 | 0.0038 | 184,088 | 4.35E-4 | 80.05 |  | G | 0.0627 | 0.0195 |
| rs12412743 | 10 | 114,045,333 | C | 0.0291 | 0.0045 | 187,095 | 2.23E-4 | 41.82 |  | C | 0.0298 | 0.0236 |
| rs970548 | 10 | 46,013,277 | C | 0.0258 | 0.0039 | 186,596 | 2.34E-4 | 43.76 |  | C | -0.0229 | 0.0202 |
| rs10761771 | 10 | 65,230,164 | C | 0.0198 | 0.0034 | 182,895 | 1.85E-4 | 33.91 |  | C | -0.0161 | 0.0176 |
| rs12740374 | 1 | 109,817,590 | T | 0.0343 | 0.0041 | 186,888 | 3.74E-4 | 69.99 |  | T | -0.0298 | 0.0214 |
| rs10501321 | 11 | 47,294,626 | C | 0.0483 | 0.0036 | 186,984 | 9.62E-4 | 180.01 |  | C | 0.0189 | 0.0191 |
| rs12145743 | 1 | 156,700,651 | G | 0.0203 | 0.0036 | 181,336 | 1.75E-4 | 31.80 |  | G | -0.0170 | 0.0187 |
| rs102275 | 11 | 61,557,803 | T | 0.0391 | 0.0035 | 187,085 | 6.67E-4 | 124.80 |  | T | -0.0141 | 0.0186 |
| rs12801636 | 11 | 65,391,317 | A | 0.0235 | 0.0042 | 187,099 | 1.67E-4 | 31.31 |  | A | -0.0171 | 0.0212 |
| rs499974 | 11 | 75,455,021 | C | 0.0263 | 0.0044 | 186,749 | 1.91E-4 | 35.73 |  | C | -0.0080 | 0.0236 |
| rs4650994 | 1 | 178,515,312 | G | 0.0210 | 0.0034 | 186,927 | 2.04E-4 | 38.15 |  | G | 0.0012 | 0.0176 |
| rs1689797 | 1 | 182,150,978 | C | 0.0358 | 0.0036 | 187,126 | 5.28E-4 | 98.89 |  | C | 0.0358 | 0.0187 |
| rs2241210 | 12 | 109,950,144 | G | 0.0332 | 0.0035 | 174,782 | 5.15E-4 | 89.98 |  | G | -0.0284 | 0.0177 |
| rs11065987 | 12 | 112,072,424 | A | 0.0222 | 0.0035 | 187,119 | 2.15E-4 | 40.23 |  | A | 0.0270 | 0.0180 |
| rs2454722 | 12 | 123,171,218 | G | 0.0351 | 0.0044 | 186,355 | 3.41E-4 | 63.64 |  | G | 0.0381 | 0.0227 |
| rs838876 | 12 | 125,259,888 | A | 0.0493 | 0.0039 | 173,066 | 9.22E-4 | 159.79 |  | A | 0.0082 | 0.0190 |
| rs7306660 | 12 | 125,327,384 | G | 0.0345 | 0.0036 | 186,939 | 4.91E-4 | 91.84 |  | G | 0.0254 | 0.0188 |
| rs4379922 | 12 | 125,351,116 | C | 0.0247 | 0.0036 | 186,203 | 2.53E-4 | 47.07 |  | C | -0.0059 | 0.0183 |
| rs2642438 | 1 | 220,970,028 | G | 0.0303 | 0.0039 | 179,439 | 3.36E-4 | 60.36 |  | G | 0.0064 | 0.0200 |
| rs11045163 | 12 | 20,463,526 | G | 0.0217 | 0.0035 | 187,075 | 2.05E-4 | 38.44 |  | G | 0.0211 | 0.0180 |
| rs4846914 | 1 | 230,295,691 | A | 0.0479 | 0.0034 | 186,995 | 1.06E-3 | 198.48 |  | A | -0.0048 | 0.0181 |
| rs3741414 | 12 | 57,844,049 | T | 0.0296 | 0.0040 | 187,090 | 2.93E-4 | 54.76 |  | T | 0.0383 | 0.0213 |
| rs12748152 | 1 | 27,138,393 | C | 0.0506 | 0.0062 | 187,057 | 3.56E-4 | 66.61 |  | C | 0.0064 | 0.0331 |
| rs4660293 | 1 | 40,028,180 | A | 0.0353 | 0.0040 | 187,027 | 4.16E-4 | 77.88 |  | A | -0.0379 | 0.0207 |
| rs4983559 | 14 | 105,277,209 | G | 0.0197 | 0.0036 | 183,672 | 1.63E-4 | 29.94 |  | G | -0.0089 | 0.0182 |
| rs492571 | 15 | 44,211,273 | T | 0.0663 | 0.0090 | 177,352 | 3.06E-4 | 54.27 |  | T | 0.0136 | 0.0430 |
| rs10468017 | 15 | 58,678,512 | T | 0.1179 | 0.0038 | 181,223 | 5.28E-3 | 962.62 |  | T | -0.0225 | 0.0196 |
| rs633695 | 15 | 58,725,839 | G | 0.0885 | 0.0054 | 92,820 | 2.89E-3 | 268.59 |  | G | -0.0008 | 0.0196 |
| rs424346 | 15 | 59,010,962 | T | 0.0679 | 0.0113 | 128,346 | 2.81E-4 | 36.11 |  | T | 0.0175 | 0.0503 |
| rs2241770 | 16 | 56,866,196 | T | 0.0989 | 0.0057 | 185,537 | 1.62E-3 | 301.05 |  | T | -0.0237 | 0.0300 |
| rs9989419 | 16 | 56,985,139 | G | 0.1473 | 0.0036 | 177,532 | 9.34E-3 | 1674.15 |  | G | 0.0107 | 0.0185 |
| rs16965220 | 16 | 57,065,121 | A | 0.0219 | 0.0037 | 185,512 | 1.89E-4 | 35.03 |  | A | 0.0003 | 0.0189 |
| rs16942887 | 16 | 67,928,042 | A | 0.0831 | 0.0051 | 185,604 | 1.43E-3 | 265.50 |  | A | -0.0200 | 0.0267 |
| rs2925979 | 16 | 81,534,790 | C | 0.0351 | 0.0037 | 185,553 | 4.85E-4 | 89.99 |  | C | 0.0074 | 0.0193 |
| rs1877031 | 17 | 37,814,080 | A | 0.0336 | 0.0036 | 185,471 | 4.69E-4 | 87.11 |  | A | -0.0128 | 0.0188 |
| rs4148005 | 17 | 66,882,466 | T | 0.0283 | 0.0036 | 184,431 | 3.35E-4 | 61.80 |  | T | 0.0064 | 0.0190 |
| rs4969178 | 17 | 76,388,202 | G | 0.0263 | 0.0035 | 185,426 | 3.04E-4 | 56.46 |  | G | 0.0623 | 0.0181 |
| rs4939883 | 18 | 47,167,214 | C | 0.0799 | 0.0045 | 185,576 | 1.70E-3 | 315.26 |  | C | -0.0229 | 0.0236 |
| rs6567160 | 18 | 57,829,135 | T | 0.0257 | 0.0041 | 185,608 | 2.12E-4 | 39.29 |  | T | -0.0171 | 0.0207 |
| rs737337 | 19 | 11,347,493 | T | 0.0565 | 0.0061 | 185,432 | 4.62E-4 | 85.79 |  | T | 0.0523 | 0.0329 |
| rs12133576 | 1 | 93,816,400 | A | 0.0243 | 0.0035 | 187,123 | 2.58E-4 | 48.20 |  | A | -0.0150 | 0.0184 |
| rs731839 | 19 | 33,899,065 | A | 0.0220 | 0.0037 | 185,498 | 1.91E-4 | 35.35 |  | A | -0.0257 | 0.0188 |
| rs2075650 | 19 | 45,395,619 | A | 0.0554 | 0.0051 | 175,421 | 6.72E-4 | 118.00 |  | A | -0.0270 | 0.0259 |
| rs2288912 | 19 | 45,449,199 | G | 0.0297 | 0.0036 | 178,910 | 3.80E-4 | 68.06 |  | G | -0.0034 | 0.0178 |
| rs103294 | 19 | 54,797,848 | T | 0.0523 | 0.0044 | 175,917 | 8.02E-4 | 141.28 |  | T | -0.0164 | 0.0226 |
| rs2278236 | 19 | 8,431,581 | A | 0.0331 | 0.0035 | 185,450 | 4.82E-4 | 89.44 |  | A | 0.0271 | 0.0177 |
| rs6031587 | 20 | 43,038,249 | C | 0.0488 | 0.0074 | 163,095 | 2.67E-4 | 43.49 |  | C | 0.0060 | 0.0342 |
| rs4465830 | 20 | 44,585,420 | A | 0.0597 | 0.0044 | 185,505 | 9.91E-4 | 184.09 |  | A | 0.0141 | 0.0227 |
| rs7607980 | 2 | 165,551,201 | C | 0.0447 | 0.0052 | 187,036 | 3.95E-4 | 73.89 |  | C | -0.0086 | 0.0262 |
| rs676210 | 2 | 21,231,524 | A | 0.0660 | 0.0040 | 187,081 | 1.45E-3 | 272.25 |  | A | -0.0471 | 0.0213 |
| rs1047891 | 2 | 211,540,507 | C | 0.0269 | 0.0039 | 182,043 | 2.61E-4 | 47.57 |  | C | -0.0034 | 0.0198 |
| rs181360 | 22 | 21,928,916 | T | 0.0376 | 0.0042 | 178,216 | 4.50E-4 | 80.14 |  | T | 0.0328 | 0.0224 |
| rs1515110 | 2 | 227,122,216 | G | 0.0323 | 0.0035 | 187,081 | 4.55E-4 | 85.17 |  | G | 0.0223 | 0.0183 |
| rs2606736 | 3 | 11,400,249 | C | 0.0246 | 0.0043 | 129,328 | 2.53E-4 | 32.73 |  | C | -0.0130 | 0.0183 |
| rs6805251 | 3 | 119,560,606 | T | 0.0200 | 0.0035 | 186,301 | 1.75E-4 | 32.65 |  | T | -0.0046 | 0.0181 |
| rs13076253 | 3 | 131,751,775 | A | 0.0283 | 0.0048 | 186,809 | 1.86E-4 | 34.76 |  | A | -0.0320 | 0.0255 |
| rs687339 | 3 | 135,932,359 | C | 0.0316 | 0.0042 | 187,105 | 3.02E-4 | 56.61 |  | C | 0.0246 | 0.0212 |
| rs2013208 | 3 | 50,129,399 | T | 0.0254 | 0.0036 | 169,708 | 2.93E-4 | 49.78 |  | T | -0.0227 | 0.0177 |
| rs13099479 | 3 | 52,677,478 | A | 0.0360 | 0.0062 | 187,132 | 1.80E-4 | 33.71 |  | A | -0.0146 | 0.0308 |
| rs2602836 | 4 | 100,014,805 | A | 0.0192 | 0.0034 | 187,102 | 1.70E-4 | 31.89 |  | A | -0.0166 | 0.0179 |
| rs13107325 | 4 | 103,188,709 | C | 0.0708 | 0.0078 | 179,316 | 4.59E-4 | 82.39 |  | C | -0.0501 | 0.0357 |
| rs10019888 | 4 | 26,062,990 | A | 0.0270 | 0.0046 | 187,077 | 1.84E-4 | 34.45 |  | A | 0.0401 | 0.0240 |
| rs3822072 | 4 | 89,741,269 | G | 0.0251 | 0.0034 | 187,115 | 2.91E-4 | 54.50 |  | G | 0.0131 | 0.0178 |
| rs6450176 | 5 | 53,298,025 | G | 0.0254 | 0.0039 | 187,132 | 2.27E-4 | 42.42 |  | G | -0.0399 | 0.0202 |
| rs1936800 | 6 | 127,436,064 | C | 0.0200 | 0.0034 | 187,111 | 1.85E-4 | 34.60 |  | C | 0.0277 | 0.0177 |
| rs3861397 | 6 | 139,828,916 | A | 0.0240 | 0.0036 | 187,084 | 2.38E-4 | 44.44 |  | A | -0.0015 | 0.0188 |
| rs9457931 | 6 | 160,929,904 | A | 0.0552 | 0.0073 | 171,669 | 3.33E-4 | 57.18 |  | A | -0.0118 | 0.0393 |
| rs1980493 | 6 | 32,363,215 | T | 0.0318 | 0.0048 | 183,275 | 2.39E-4 | 43.89 |  | T | 0.0142 | 0.0247 |
| rs205262 | 6 | 34,563,164 | A | 0.0283 | 0.0039 | 181,707 | 2.90E-4 | 52.65 |  | A | 0.0214 | 0.0199 |
| rs998584 | 6 | 43,757,896 | C | 0.0260 | 0.0038 | 183,791 | 2.55E-4 | 46.81 |  | C | -0.0011 | 0.0187 |
| rs11765979 | 7 | 130,445,877 | C | 0.0412 | 0.0048 | 94,311 | 7.81E-4 | 73.67 |  | C | 0.0282 | 0.0177 |
| rs17173637 | 7 | 150,529,449 | T | 0.0363 | 0.0057 | 183,901 | 2.20E-4 | 40.56 |  | T | -0.0202 | 0.0304 |
| rs4142995 | 7 | 17,919,258 | G | 0.0263 | 0.0037 | 165,161 | 3.06E-4 | 50.52 |  | G | -0.0026 | 0.0180 |
| rs4917014 | 7 | 50,305,863 | G | 0.0222 | 0.0036 | 186,868 | 2.03E-4 | 38.03 |  | G | 0.0080 | 0.0188 |
| rs702485 | 7 | 6,449,272 | G | 0.0243 | 0.0034 | 186,974 | 2.73E-4 | 51.08 |  | G | -0.0037 | 0.0178 |
| rs17145738 | 7 | 72,982,874 | T | 0.0408 | 0.0053 | 184,971 | 3.20E-4 | 59.26 |  | T | 0.0142 | 0.0280 |
| rs2293889 | 8 | 116,599,199 | G | 0.0312 | 0.0035 | 180,102 | 4.41E-4 | 79.46 |  | G | 0.0175 | 0.0179 |
| rs10808546 | 8 | 126,495,818 | T | 0.0409 | 0.0034 | 185,835 | 7.78E-4 | 144.71 |  | T | -0.0012 | 0.0177 |
| rs10087900 | 8 | 144,303,418 | G | 0.0231 | 0.0036 | 183,672 | 2.24E-4 | 41.17 |  | G | 0.0185 | 0.0178 |
| rs1866956 | 8 | 19,748,921 | T | 0.0217 | 0.0037 | 187,031 | 1.84E-4 | 34.40 |  | T | 0.0231 | 0.0192 |
| rs13702 | 8 | 19,824,492 | C | 0.1058 | 0.0038 | 187,044 | 4.13E-3 | 775.17 |  | C | -0.0067 | 0.0193 |
| rs4240624 | 8 | 9,184,231 | A | 0.0818 | 0.0058 | 185,696 | 1.07E-3 | 198.91 |  | A | -0.0231 | 0.0320 |
| rs2066714 | 9 | 107,586,753 | C | 0.0453 | 0.0071 | 93,528 | 4.35E-4 | 40.71 |  | C | -0.0031 | 0.0263 |
| rs11789603 | 9 | 107,647,019 | T | 0.0600 | 0.0060 | 184,432 | 5.42E-4 | 100.00 |  | T | -0.0261 | 0.0291 |
| rs1883025 | 9 | 107,664,301 | C | 0.0698 | 0.0041 | 186,365 | 1.55E-3 | 289.83 |  | C | -0.0325 | 0.0204 |
| rs686030 | 9 | 15,304,782 | A | 0.0550 | 0.0049 | 187,035 | 6.73E-4 | 125.99 |  | A | 0.0634 | 0.0255 |
| rs7112577 | 11 | 117,044,603 | G | 0.0826 | 0.0129 | 89,235 | 4.59E-4 | 41.00 |  | G | -0.0829 | 0.0469 |
| rs964184 | 11 | 116,648,917 | C | 0.1065 | 0.0071 | 94,289 | 2.38E-3 | 225.00 |  | C | -0.0296 | 0.0257 |

In the Allele column, the first one is the effect allele. HDL: high density lipoprotein, ALS: amyotrophic lateral sclerosis, Chr: chromosome, SNP: single-nucleotide polymorphism, Beta: SNP effect size, SE: standard error; *N* and *F* are respectively the sample size and *F* statistic for each SNP. These instruments collectively explain 6.15% HDL variation (i.e. proportion of variance explained [PVE] by SNPs = 6.15% using the method introduced in ), we compute the *F* statistic for each instrument via $F_{i} = \text{PVE}_{i}\text{(}N_{i} -1-k\text{)/}(k-k\times\text{PVE}_{i})$, where *N_i_* is the sample size for instrument *i* and *k* is the number of total instruments. All the *F* statistics are larger than 10 (ranging from 29.94 to 1674.15). The overall *F* statistic for the 88 instrumental variables is 132.02 (PVE = 6.15%, *k* = 88 and *N* = 177,372), suggesting that the selected genetic variants have sufficient strong effects for instrument and the bias of weak instrument is unlikely.

**Table S3.** Index SNPs used as instruments for LDL and the effects, standard errors and association strength for ASL in the European population

| SNP | CHR | Position (GRCh37) | LDL | | | | | |  | ASL | | |
| --- | --- | --- | --- | --- | --- | --- | --- | --- | --- | --- | --- | --- |
|  |  |  | Allele | Beta | SE | *N* | PVE | *F* |  | Allele | Beta | SE |
| rs2419604 | 10 | 113,944,271 | A | 0.0302 | 0.0040 | 172,807 | 3.30E-4 | 57.00 |  | A | 0.0646 | 0.0195 |
| rs646776 | 1 | 109,818,530 | T | 0.1602 | 0.0044 | 173,021 | 7.60E-3 | 1325.61 |  | T | 0.0311 | 0.0214 |
| rs10893499 | 11 | 126,241,979 | A | 0.0521 | 0.0053 | 172,980 | 5.58E-4 | 96.63 |  | A | -0.0306 | 0.0258 |
| rs10832962 | 11 | 18,656,271 | T | 0.0320 | 0.0040 | 172,920 | 3.70E-4 | 64.00 |  | T | 0.0118 | 0.0202 |
| rs267733 | 1 | 150,958,836 | A | 0.0331 | 0.0053 | 164,562 | 2.37E-4 | 39.00 |  | A | -0.0402 | 0.0242 |
| rs174583 | 11 | 61,609,750 | C | 0.0522 | 0.0038 | 172,982 | 1.09E-3 | 188.70 |  | C | -0.0214 | 0.0187 |
| rs3184504 | 12 | 111,884,608 | C | 0.0268 | 0.0038 | 164,996 | 3.01E-4 | 49.74 |  | C | 0.0467 | 0.0177 |
| rs1169288 | 12 | 121,416,650 | C | 0.0375 | 0.0040 | 163,086 | 5.39E-4 | 87.89 |  | C | 0.0007 | 0.0192 |
| rs2642438 | 1 | 220,970,028 | G | 0.0352 | 0.0042 | 165,470 | 4.24E-4 | 70.24 |  | G | 0.0064 | 0.0200 |
| rs2587534 | 1 | 234,849,339 | A | 0.0391 | 0.0037 | 172,966 | 6.45E-4 | 111.67 |  | A | -0.0135 | 0.0178 |
| rs10903129 | 1 | 25,768,937 | G | 0.0328 | 0.0037 | 169,920 | 4.62E-4 | 78.58 |  | G | 0.0098 | 0.0177 |
| rs12748152 | 1 | 27,138,393 | T | 0.0499 | 0.0066 | 172,988 | 3.30E-4 | 57.16 |  | T | -0.0064 | 0.0331 |
| rs4942486 | 13 | 32,953,388 | T | 0.0243 | 0.0037 | 171,930 | 2.51E-4 | 43.13 |  | T | -0.0294 | 0.0177 |
| rs8017377 | 14 | 24,883,887 | A | 0.0303 | 0.0038 | 172,866 | 3.68E-4 | 63.58 |  | A | -0.0124 | 0.0177 |
| rs11591147 | 1 | 55,505,647 | G | 0.4970 | 0.0180 | 77,417 | 9.75E-3 | 762.35 |  | G | -0.0755 | 0.0727 |
| rs2902875 | 1 | 55,695,535 | T | 0.0756 | 0.0126 | 89,883 | 4.00E-4 | 36.00 |  | T | 0.0101 | 0.0444 |
| rs7551981 | 1 | 55,719,166 | T | 0.0472 | 0.0038 | 173,021 | 8.91E-4 | 154.28 |  | T | 0.0257 | 0.0182 |
| rs7534572 | 1 | 62,999,675 | G | 0.0407 | 0.0058 | 75,145 | 6.55E-4 | 49.24 |  | G | -0.0017 | 0.0188 |
| rs247616 | 16 | 56,989,590 | C | 0.0547 | 0.0041 | 171,458 | 1.04E-3 | 177.99 |  | C | 0.0135 | 0.0192 |
| rs2000999 | 16 | 72,108,093 | A | 0.0650 | 0.0046 | 171,510 | 1.16E-3 | 199.67 |  | A | 0.0049 | 0.0220 |
| rs6504872 | 17 | 45,438,952 | T | 0.0274 | 0.0037 | 171,519 | 3.20E-4 | 54.84 |  | T | -0.0351 | 0.0177 |
| rs1801689 | 17 | 64,210,580 | C | 0.1028 | 0.0139 | 111,143 | 4.92E-4 | 54.70 |  | C | -0.0304 | 0.0564 |
| rs2886232 | 17 | 67,150,176 | T | 0.0451 | 0.0064 | 162,498 | 3.06E-4 | 49.66 |  | T | -0.0025 | 0.0286 |
| rs314253 | 17 | 7,091,650 | T | 0.0242 | 0.0038 | 169,706 | 2.39E-4 | 40.56 |  | T | -0.0099 | 0.0186 |
| rs6511720 | 19 | 11,202,306 | G | 0.2209 | 0.0061 | 170,608 | 7.63E-3 | 1311.37 |  | G | 0.0546 | 0.0275 |
| rs2228603 | 19 | 19,329,924 | C | 0.1040 | 0.0072 | 158,643 | 1.31E-3 | 208.64 |  | C | -0.0041 | 0.0356 |
| rs4970712 | 1 | 92,993,547 | C | 0.0339 | 0.0044 | 173,036 | 3.43E-4 | 59.36 |  | C | -0.0038 | 0.0219 |
| rs2965157 | 19 | 45,176,340 | T | 0.1886 | 0.0112 | 170,260 | 1.66E-3 | 283.56 |  | T | 0.0106 | 0.0584 |
| rs7254892 | 19 | 45,389,596 | G | 0.4853 | 0.0119 | 139,198 | 1.18E-2 | 1663.11 |  | G | 0.0696 | 0.0502 |
| rs75687619 | 19 | 45,399,344 | T | 0.1735 | 0.0161 | 82,004 | 1.41E-3 | 116.13 |  | T | 0.0238 | 0.0596 |
| rs12721109 | 19 | 45,447,221 | G | 0.4462 | 0.0183 | 99,409 | 5.94E-3 | 594.50 |  | G | 0.0468 | 0.0662 |
| rs676388 | 19 | 49,211,969 | C | 0.0265 | 0.0039 | 166,830 | 2.77E-4 | 46.17 |  | C | 0.0124 | 0.0177 |
| rs364585 | 20 | 12,962,718 | G | 0.0249 | 0.0038 | 171,526 | 2.50E-4 | 42.94 |  | G | 0.0134 | 0.0181 |
| rs2328223 | 20 | 17,845,921 | C | 0.0299 | 0.0050 | 170,762 | 2.09E-4 | 35.76 |  | C | -0.0109 | 0.0220 |
| rs6016373 | 20 | 39,154,095 | A | 0.0349 | 0.0037 | 171,559 | 5.18E-4 | 88.97 |  | A | 0.0099 | 0.0182 |
| rs6065311 | 20 | 39,724,338 | C | 0.0417 | 0.0036 | 171,333 | 7.83E-4 | 134.17 |  | C | 0.0417 | 0.0176 |
| rs1800961 | 20 | 43,042,364 | C | 0.0685 | 0.0106 | 142,698 | 2.93E-4 | 41.76 |  | C | -0.0544 | 0.0505 |
| rs10490626 | 2 | 118,835,841 | G | 0.0508 | 0.0069 | 173,044 | 3.13E-4 | 54.20 |  | G | 0.0087 | 0.0337 |
| rs2030746 | 2 | 121,309,488 | T | 0.0214 | 0.0038 | 173,024 | 1.83E-4 | 31.71 |  | T | -0.0069 | 0.0180 |
| rs16831243 | 2 | 135,762,344 | T | 0.0378 | 0.0055 | 162,945 | 2.90E-4 | 47.23 |  | T | 0.0158 | 0.0249 |
| rs10195252 | 2 | 165,513,091 | T | 0.0238 | 0.0039 | 157,208 | 2.37E-4 | 37.24 |  | T | 0.0181 | 0.0180 |
| rs1367117 | 2 | 21,263,900 | A | 0.1186 | 0.0040 | 173,007 | 5.06E-3 | 879.11 |  | A | 0.0339 | 0.0193 |
| rs72902576 | 2 | 21,275,480 | T | 0.0933 | 0.0133 | 82,068 | 5.99E-4 | 49.21 |  | T | 0.0284 | 0.0480 |
| rs1250229 | 2 | 216,304,384 | C | 0.0243 | 0.0042 | 173,032 | 1.93E-4 | 33.47 |  | C | -0.0334 | 0.0205 |
| rs5763662 | 22 | 30,378,703 | T | 0.0767 | 0.0121 | 162,777 | 2.47E-4 | 40.18 |  | T | 0.1082 | 0.0599 |
| rs11563251 | 2 | 234,679,384 | T | 0.0345 | 0.0062 | 172,855 | 1.79E-4 | 30.96 |  | T | -0.0367 | 0.0292 |
| rs4253776 | 22 | 46,629,479 | G | 0.0311 | 0.0059 | 171,071 | 1.62E-4 | 27.79 |  | G | -0.0156 | 0.0281 |
| rs6544713 | 2 | 44,073,881 | T | 0.0806 | 0.0041 | 172,940 | 2.23E-3 | 386.45 |  | T | 0.0182 | 0.0192 |
| rs6709904 | 2 | 44,080,324 | A | 0.0550 | 0.0085 | 89,852 | 4.66E-4 | 41.87 |  | A | -0.0178 | 0.0278 |
| rs2710642 | 2 | 63,149,557 | A | 0.0239 | 0.0038 | 172,994 | 2.29E-4 | 39.56 |  | A | 0.0098 | 0.0186 |
| rs9875338 | 3 | 12,296,469 | G | 0.0270 | 0.0037 | 172,895 | 3.08E-4 | 53.25 |  | G | 0.0042 | 0.0180 |
| rs17404153 | 3 | 132,163,200 | G | 0.0336 | 0.0054 | 172,898 | 2.24E-4 | 38.72 |  | G | -0.0282 | 0.0269 |
| rs7640978 | 3 | 32,533,010 | C | 0.0392 | 0.0069 | 172,228 | 1.87E-4 | 32.28 |  | C | 0.0166 | 0.0313 |
| rs6818397 | 4 | 3,434,885 | T | 0.0224 | 0.0040 | 172,685 | 1.82E-4 | 31.36 |  | T | 0.0001 | 0.0184 |
| rs4530754 | 5 | 122,855,416 | A | 0.0275 | 0.0036 | 173,003 | 3.37E-4 | 58.35 |  | A | 0.0062 | 0.0178 |
| rs6882076 | 5 | 156,390,297 | C | 0.0456 | 0.0038 | 173,006 | 8.32E-4 | 144.00 |  | C | 0.0062 | 0.0183 |
| rs12916 | 5 | 74,656,539 | C | 0.0733 | 0.0038 | 168,357 | 2.21E-3 | 372.08 |  | C | -0.0120 | 0.0180 |
| rs6909746 | 6 | 116,352,750 | C | 0.0263 | 0.0037 | 170,097 | 2.97E-4 | 50.52 |  | C | 0.0169 | 0.0178 |
| rs112201728 | 6 | 160,551,486 | T | 0.0675 | 0.0104 | 83,124 | 5.07E-4 | 42.12 |  | T | 0.0014 | 0.0348 |
| rs1564348 | 6 | 160,578,860 | C | 0.0481 | 0.0050 | 172,989 | 5.35E-4 | 92.54 |  | C | -0.0128 | 0.0242 |
| rs16891156 | 6 | 160,608,804 | C | 0.0965 | 0.0171 | 90,466 | 3.52E-4 | 31.85 |  | C | -0.0006 | 0.0680 |
| rs2315065 | 6 | 161,108,144 | A | 0.1102 | 0.0158 | 67,446 | 7.21E-4 | 48.64 |  | A | 0.0479 | 0.0332 |
| rs3757354 | 6 | 16,127,407 | C | 0.0382 | 0.0044 | 172,987 | 4.36E-4 | 75.37 |  | C | -0.0123 | 0.0215 |
| rs13206249 | 6 | 16,175,022 | G | 0.0378 | 0.0062 | 87,149 | 4.26E-4 | 37.17 |  | G | 0.0255 | 0.0218 |
| rs1408272 | 6 | 25,842,951 | T | 0.0520 | 0.0083 | 167,888 | 2.34E-4 | 39.25 |  | T | 0.0251 | 0.0374 |
| rs10947332 | 6 | 32,677,440 | A | 0.0504 | 0.0056 | 169,263 | 4.78E-4 | 81.00 |  | A | 0.0194 | 0.0274 |
| rs2390536 | 7 | 21,485,397 | A | 0.0223 | 0.0038 | 172,981 | 1.99E-4 | 34.44 |  | A | 0.0167 | 0.0185 |
| rs4722551 | 7 | 25,991,826 | C | 0.0391 | 0.0049 | 172,946 | 3.68E-4 | 63.67 |  | C | -0.0123 | 0.0238 |
| rs2073547 | 7 | 44,582,331 | G | 0.0485 | 0.0049 | 169,889 | 5.76E-4 | 97.97 |  | G | 0.0481 | 0.0231 |
| rs2737252 | 8 | 116,663,898 | G | 0.0314 | 0.0041 | 172,950 | 3.39E-4 | 58.65 |  | G | 0.0386 | 0.0199 |
| rs2954029 | 8 | 126,490,972 | A | 0.0564 | 0.0036 | 172,963 | 1.42E-3 | 245.44 |  | A | -0.0044 | 0.0177 |
| rs7832643 | 8 | 145,022,657 | T | 0.0339 | 0.0038 | 164,854 | 4.83E-4 | 79.58 |  | T | -0.0190 | 0.0184 |
| rs13277801 | 8 | 59,353,534 | C | 0.0338 | 0.0038 | 173,010 | 4.57E-4 | 79.12 |  | C | -0.0077 | 0.0185 |
| rs9987289 | 8 | 9,183,358 | G | 0.0714 | 0.0066 | 160,102 | 7.30E-4 | 117.03 |  | G | -0.0231 | 0.0318 |
| rs1883025 | 9 | 107,664,301 | C | 0.0296 | 0.0044 | 172,330 | 2.63E-4 | 45.26 |  | C | -0.0325 | 0.0204 |
| rs579459 | 9 | 136,154,168 | C | 0.0665 | 0.0045 | 172,706 | 1.26E-3 | 218.38 |  | C | 0.0402 | 0.0215 |
| rs3780181 | 9 | 2,640,759 | A | 0.0445 | 0.0074 | 171,976 | 2.10E-4 | 36.16 |  | A | 0.0771 | 0.0359 |
| rs964184 | 11 | 116,648,917 | G | 0.0855 | 0.0078 | 89,866 | 1.34E-3 | 120.15 |  | G | 0.0296 | 0.0257 |

In the Allele column, the first one is the effect allele. LDL: low density lipoprotein, ALS: amyotrophic lateral sclerosis, Chr: chromosome, SNP: single-nucleotide polymorphism, Beta: SNP effect size, SE: standard error; *N* and *F* are respectively the sample size and *F* statistic for each SNP. These instruments collectively explain 8.75% HDL variation (i.e. proportion of variance explained [PVE] by SNPs = 8.75%), we compute the *F* statistic for each instrument via $F_{i} = \text{PVE}_{i}\text{(}N_{i} -1-k\text{)/}(k-k\times\text{PVE}_{i})$, where *N_i_* is the sample size for instrument *i* and *k* is the number of total instruments. All the *F* statistics are larger than 10 (ranging from 27.79 to 1663.11). The overall *F* statistic for the 78 instrumental variables is 191.16 (with PVE = 8.75%, *k* = 78 and *N* = 155,571), suggesting that the selected genetic variants have sufficient strong effects for instrument and the bias of weak instrument is unlikely.

**Table S4.** Index SNPs used as instruments for TC and the effects, standard errors and association strength for ASL in the European population

| SNP | CHR | Position (GRCh37) | TC | | | | | |  | ASL | | |
| --- | --- | --- | --- | --- | --- | --- | --- | --- | --- | --- | --- | --- |
|  |  |  | Allele | Beta | SE | *N* | PVE | *F* |  | Allele | Beta | SE |
| rs2255141 | 10 | 113,933,886 | A | 0.0314 | 0.0039 | 187,266 | 3.46E-4 | 64.82 |  | A | 0.0635 | 0.0195 |
| rs12412743 | 10 | 114,045,333 | C | 0.0298 | 0.0047 | 187,282 | 2.15E-4 | 40.20 |  | C | 0.0298 | 0.0236 |
| rs10904908 | 10 | 17,260,290 | G | 0.0250 | 0.0036 | 187,112 | 2.58E-4 | 48.22 |  | G | -0.0052 | 0.0180 |
| rs10900221 | 10 | 45,988,597 | A | 0.0255 | 0.0041 | 186,785 | 2.07E-4 | 38.68 |  | A | -0.0269 | 0.0204 |
| rs646776 | 1 | 109,818,530 | T | 0.1272 | 0.0042 | 187,288 | 4.87E-3 | 917.21 |  | T | 0.0311 | 0.0214 |
| rs11220462 | 11 | 126,243,952 | A | 0.0474 | 0.0058 | 156,953 | 4.25E-4 | 66.79 |  | A | -0.0319 | 0.0258 |
| rs10832962 | 11 | 18,656,271 | T | 0.0315 | 0.0039 | 187,161 | 3.48E-4 | 65.24 |  | T | 0.0118 | 0.0202 |
| rs4752805 | 11 | 48,018,355 | G | 0.0251 | 0.0041 | 187,233 | 2.00E-4 | 37.48 |  | G | 0.0314 | 0.0202 |
| rs1535 | 11 | 61,597,972 | A | 0.0497 | 0.0037 | 182,527 | 9.88E-4 | 180.43 |  | A | -0.0139 | 0.0187 |
| rs3184504 | 12 | 111,884,608 | C | 0.0318 | 0.0037 | 177,714 | 4.15E-4 | 73.87 |  | C | 0.0467 | 0.0177 |
| rs2244608 | 12 | 121,416,988 | G | 0.0313 | 0.0037 | 187,223 | 3.82E-4 | 71.56 |  | G | 0.0028 | 0.0190 |
| rs10773003 | 12 | 123,775,127 | A | 0.0369 | 0.0058 | 187,101 | 2.16E-4 | 40.48 |  | A | 0.0131 | 0.0306 |
| rs2642438 | 1 | 220,970,028 | G | 0.0370 | 0.0040 | 179,599 | 4.76E-4 | 85.56 |  | G | 0.0064 | 0.0200 |
| rs558971 | 1 | 234,853,406 | G | 0.0398 | 0.0036 | 187,254 | 6.52E-4 | 122.22 |  | G | -0.0153 | 0.0178 |
| rs11802413 | 1 | 25,760,920 | T | 0.0287 | 0.0035 | 187,138 | 3.59E-4 | 67.24 |  | T | 0.0099 | 0.0177 |
| rs6573778 | 14 | 24,872,209 | T | 0.0263 | 0.0039 | 187,079 | 2.43E-4 | 45.48 |  | T | -0.0056 | 0.0180 |
| rs11591147 | 1 | 55,505,647 | G | 0.3341 | 0.0173 | 85,729 | 4.33E-3 | 372.95 |  | G | -0.0755 | 0.0727 |
| rs2902875 | 1 | 55,695,535 | T | 0.0707 | 0.0123 | 94,590 | 3.49E-4 | 33.04 |  | T | 0.0101 | 0.0444 |
| rs7551981 | 1 | 55,719,166 | T | 0.0358 | 0.0037 | 187,282 | 5.00E-4 | 93.62 |  | T | 0.0257 | 0.0182 |
| rs10468017 | 15 | 58,678,512 | T | 0.0617 | 0.0040 | 181,378 | 1.31E-3 | 237.93 |  | T | -0.0225 | 0.0196 |
| rs633695 | 15 | 58,725,839 | G | 0.0433 | 0.0058 | 93,067 | 5.98E-4 | 55.73 |  | G | -0.0008 | 0.0196 |
| rs7534572 | 1 | 62,999,675 | G | 0.0629 | 0.0055 | 83,151 | 1.57E-3 | 130.79 |  | G | -0.0017 | 0.0188 |
| rs247616 | 16 | 56,989,590 | T | 0.0499 | 0.0040 | 185,621 | 8.38E-4 | 155.62 |  | T | -0.0135 | 0.0192 |
| rs2000999 | 16 | 72,108,093 | A | 0.0617 | 0.0044 | 185,692 | 1.06E-3 | 196.63 |  | A | 0.0049 | 0.0220 |
| rs6504872 | 17 | 45,438,952 | T | 0.0250 | 0.0035 | 185,712 | 2.75E-4 | 51.02 |  | T | -0.0351 | 0.0177 |
| rs2886232 | 17 | 67,150,176 | T | 0.0358 | 0.0062 | 176,571 | 1.89E-4 | 33.34 |  | T | -0.0025 | 0.0286 |
| rs314253 | 17 | 7,091,650 | T | 0.0233 | 0.0037 | 183,868 | 2.16E-4 | 39.66 |  | T | -0.0099 | 0.0186 |
| rs2156552 | 18 | 47,181,668 | T | 0.0570 | 0.0047 | 183,439 | 8.01E-4 | 147.08 |  | T | -0.0265 | 0.0238 |
| rs6511720 | 19 | 11,202,306 | G | 0.1851 | 0.0059 | 184,764 | 5.30E-3 | 984.25 |  | G | 0.0546 | 0.0275 |
| rs2228603 | 19 | 19,329,924 | C | 0.1217 | 0.0069 | 170,510 | 1.82E-3 | 311.08 |  | C | -0.0041 | 0.0356 |
| rs6603981 | 1 | 92,993,807 | T | 0.0351 | 0.0043 | 187,329 | 3.56E-4 | 66.63 |  | T | -0.0040 | 0.0219 |
| rs8103315 | 19 | 45,254,168 | A | 0.0422 | 0.0055 | 156,370 | 3.76E-4 | 58.87 |  | A | -0.0141 | 0.0266 |
| rs75687619 | 19 | 45,399,344 | T | 0.1592 | 0.0153 | 91,544 | 1.18E-3 | 108.27 |  | T | 0.0238 | 0.0596 |
| rs7412 | 19 | 45,412,079 | C | 0.3736 | 0.0096 | 92,046 | 1.62E-2 | 1514.47 |  | C | 0.0114 | 0.0340 |
| rs281393 | 19 | 49,224,484 | C | 0.0322 | 0.0055 | 93,067 | 3.68E-4 | 34.27 |  | C | -0.0048 | 0.0185 |
| rs2277862 | 20 | 34,152,782 | C | 0.0349 | 0.0052 | 185,738 | 2.42E-4 | 45.04 |  | C | 0.0015 | 0.0249 |
| rs6016373 | 20 | 39,154,095 | A | 0.0319 | 0.0036 | 185,730 | 4.23E-4 | 78.52 |  | A | 0.0099 | 0.0182 |
| rs2235367 | 20 | 39,830,122 | G | 0.0357 | 0.0035 | 185,691 | 5.60E-4 | 104.04 |  | G | 0.0428 | 0.0176 |
| rs1800961 | 20 | 43,042,364 | C | 0.1062 | 0.0101 | 156,406 | 7.06E-4 | 110.56 |  | C | -0.0544 | 0.0505 |
| rs17526895 | 2 | 118,815,958 | A | 0.0420 | 0.0067 | 184,199 | 2.13E-4 | 39.30 |  | A | 0.0139 | 0.0337 |
| rs2030746 | 2 | 121,309,488 | T | 0.0199 | 0.0037 | 187,289 | 1.54E-4 | 28.93 |  | T | -0.0069 | 0.0180 |
| rs4988235 | 2 | 136,608,646 | G | 0.0308 | 0.0040 | 183,761 | 3.23E-4 | 59.29 |  | G | 0.0220 | 0.0196 |
| rs2287623 | 2 | 169,830,155 | G | 0.0273 | 0.0036 | 184,257 | 3.12E-4 | 57.51 |  | G | -0.0398 | 0.0182 |
| rs11694172 | 2 | 203,532,304 | G | 0.0277 | 0.0041 | 187,092 | 2.44E-4 | 45.64 |  | G | 0.0116 | 0.0202 |
| rs9306897 | 2 | 21,138,066 | T | 0.0488 | 0.0037 | 185,808 | 9.35E-4 | 173.95 |  | T | 0.0120 | 0.0191 |
| rs515135 | 2 | 21,286,057 | C | 0.1238 | 0.0046 | 187,291 | 3.85E-3 | 724.30 |  | C | 0.0289 | 0.0229 |
| rs181360 | 22 | 21,928,916 | T | 0.0278 | 0.0043 | 178,322 | 2.34E-4 | 41.80 |  | T | 0.0328 | 0.0224 |
| rs138777 | 22 | 35,711,098 | A | 0.0214 | 0.0037 | 185,274 | 1.81E-4 | 33.45 |  | A | 0.0353 | 0.0186 |
| rs11563251 | 2 | 234,679,384 | T | 0.0368 | 0.0059 | 187,107 | 2.08E-4 | 38.90 |  | T | -0.0367 | 0.0292 |
| rs4253772 | 22 | 46,627,603 | T | 0.0322 | 0.0058 | 185,188 | 1.66E-4 | 30.82 |  | T | -0.0146 | 0.0286 |
| rs780093 | 2 | 27,742,603 | T | 0.0515 | 0.0036 | 186,446 | 1.10E-3 | 204.65 |  | T | 0.0162 | 0.0180 |
| rs6544713 | 2 | 44,073,881 | T | 0.0773 | 0.0040 | 187,199 | 1.99E-3 | 373.45 |  | T | 0.0182 | 0.0192 |
| rs6709904 | 2 | 44,080,324 | A | 0.0545 | 0.0083 | 94,558 | 4.56E-4 | 43.11 |  | A | -0.0178 | 0.0278 |
| rs7616006 | 3 | 12,267,648 | A | 0.0315 | 0.0036 | 187,246 | 4.09E-4 | 76.56 |  | A | 0.0120 | 0.0179 |
| rs7640978 | 3 | 32,533,010 | C | 0.0376 | 0.0066 | 186,485 | 1.74E-4 | 32.46 |  | C | 0.0166 | 0.0313 |
| rs13315871 | 3 | 58,381,287 | G | 0.0355 | 0.0061 | 187,287 | 1.81E-4 | 33.87 |  | G | 0.0210 | 0.0306 |
| rs6818397 | 4 | 3,434,885 | T | 0.0254 | 0.0039 | 186,903 | 2.27E-4 | 42.42 |  | T | 0.0001 | 0.0184 |
| rs4530754 | 5 | 122,855,416 | A | 0.0228 | 0.0035 | 187,272 | 2.27E-4 | 42.44 |  | A | 0.0062 | 0.0178 |
| rs6882076 | 5 | 156,390,297 | C | 0.0508 | 0.0037 | 187,270 | 1.01E-3 | 188.50 |  | C | 0.0062 | 0.0183 |
| rs12916 | 5 | 74,656,539 | C | 0.0684 | 0.0036 | 182,530 | 1.97E-3 | 361.00 |  | C | -0.0120 | 0.0180 |
| rs11153594 | 6 | 116,354,591 | C | 0.0290 | 0.0036 | 187,230 | 3.46E-4 | 64.89 |  | C | 0.0164 | 0.0178 |
| rs9376090 | 6 | 135,411,228 | T | 0.0254 | 0.0040 | 187,263 | 2.15E-4 | 40.32 |  | T | -0.0066 | 0.0204 |
| rs112201728 | 6 | 160,551,486 | T | 0.0581 | 0.0099 | 92,668 | 3.72E-4 | 34.44 |  | T | 0.0014 | 0.0348 |
| rs11753995 | 6 | 160,575,366 | A | 0.0489 | 0.0048 | 187,264 | 5.54E-4 | 103.78 |  | A | -0.0135 | 0.0241 |
| rs2315065 | 6 | 161,108,144 | A | 0.1102 | 0.0158 | 68,430 | 7.10E-4 | 48.64 |  | A | 0.0479 | 0.0332 |
| rs3757354 | 6 | 16,127,407 | C | 0.0348 | 0.0042 | 187,247 | 3.67E-4 | 68.65 |  | C | -0.0123 | 0.0215 |
| rs1800562 | 6 | 26,093,141 | G | 0.0565 | 0.0077 | 185,469 | 2.90E-4 | 53.84 |  | G | 0.0021 | 0.0378 |
| rs9391858 | 6 | 32,341,398 | G | 0.0495 | 0.0050 | 176,743 | 5.54E-4 | 98.01 |  | G | 0.0079 | 0.0251 |
| rs9272775 | 6 | 32,610,257 | C | 0.0317 | 0.0055 | 89,534 | 3.71E-4 | 33.22 |  | C | -0.0210 | 0.0202 |
| rs2814982 | 6 | 34,546,560 | C | 0.0441 | 0.0057 | 187,263 | 3.20E-4 | 59.86 |  | C | 0.0133 | 0.0284 |
| rs1997243 | 7 | 1,083,777 | G | 0.0332 | 0.0050 | 183,314 | 2.40E-4 | 44.09 |  | G | 0.0172 | 0.0243 |
| rs12670798 | 7 | 21,607,352 | C | 0.0364 | 0.0041 | 187,287 | 4.21E-4 | 78.82 |  | C | 0.0026 | 0.0211 |
| rs2073547 | 7 | 44,582,331 | G | 0.0456 | 0.0047 | 184,098 | 5.11E-4 | 94.13 |  | G | 0.0481 | 0.0231 |
| rs2737252 | 8 | 116,663,898 | G | 0.0331 | 0.0039 | 187,202 | 3.85E-4 | 72.03 |  | G | 0.0386 | 0.0199 |
| rs2954029 | 8 | 126,490,972 | A | 0.0622 | 0.0035 | 187,216 | 1.68E-3 | 315.82 |  | A | -0.0044 | 0.0177 |
| rs7832643 | 8 | 145,022,657 | T | 0.0289 | 0.0037 | 178,980 | 3.41E-4 | 61.01 |  | T | -0.0190 | 0.0184 |
| rs10088180 | 8 | 18,228,116 | A | 0.0228 | 0.0040 | 187,142 | 1.74E-4 | 32.49 |  | A | 0.0092 | 0.0196 |
| rs4738684 | 8 | 59,393,273 | A | 0.0392 | 0.0037 | 187,285 | 5.99E-4 | 112.24 |  | A | -0.0170 | 0.0186 |
| rs9987289 | 8 | 9,183,358 | G | 0.0842 | 0.0063 | 173,502 | 1.03E-3 | 178.62 |  | G | -0.0231 | 0.0318 |
| rs2066714 | 9 | 107,586,753 | C | 0.0442 | 0.0076 | 93,811 | 3.60E-4 | 33.82 |  | C | -0.0031 | 0.0263 |
| rs11789603 | 9 | 107,647,019 | T | 0.0427 | 0.0062 | 186,565 | 2.54E-4 | 47.43 |  | T | -0.0261 | 0.0291 |
| rs1883025 | 9 | 107,664,301 | C | 0.0671 | 0.0042 | 186,557 | 1.37E-3 | 255.24 |  | C | -0.0325 | 0.0204 |
| rs579459 | 9 | 136,154,168 | C | 0.0620 | 0.0044 | 186,925 | 1.06E-3 | 198.55 |  | C | 0.0402 | 0.0215 |
| rs581080 | 9 | 15,305,378 | C | 0.0377 | 0.0047 | 187,121 | 3.44E-4 | 64.34 |  | C | 0.0291 | 0.0233 |
| rs3780181 | 9 | 2,640,759 | A | 0.0442 | 0.0071 | 186,134 | 2.08E-4 | 38.75 |  | A | 0.0771 | 0.0359 |
| rs964184 | 11 | 116,648,917 | G | 0.1214 | 0.0076 | 94,573 | 2.69E-3 | 255.15 |  | G | 0.0296 | 0.0257 |

In the Allele column, the first one is the effect allele. TC: total cholesterol, ALS: amyotrophic lateral sclerosis, Chr: chromosome, SNP: single-nucleotide polymorphism, Beta: SNP effect size, SE: standard error; *N* and *F* are respectively the sample size and *F* statistic for each SNP. These instruments collectively explain 8.05% HDL variation (i.e. proportion of variance explained [PVE] by SNPs = 8.05%), we compute the *F* statistic for each instrument via $F_{i} = \text{PVE}_{i}\text{(}N_{i} -1-k\text{)/}(k-k\times\text{PVE}_{i})$, where *N_i_* is the sample size for instrument *i* and *k* is the number of total instruments. All the *F* statistics are larger than 10 (ranging from 28.93 to 1514.47). The overall *F* statistic for the 86 instrumental variables is 171.96 (with PVE = 8.05%, *k* = 86 and *N* = 169,693), suggesting that the selected genetic variants have sufficient strong effects for instrument and the bias of weak instrument is unlikely.

**Table S5.** Index SNPs used as instruments for TG and their effects, standard errors and association strength for ASL in the European population

| SNP | Chr | Position (GRCh37) | TG | | | | | |  | ASL | | |
| --- | --- | --- | --- | --- | --- | --- | --- | --- | --- | --- | --- | --- |
|  |  |  | Allele | Beta | SE | *N* | PVE | *F* |  | Allele | Beta | SE |
| rs2250802 | 10 | 113,921,354 | A | 0.0230 | 0.0037 | 174,734 | 2.21E-4 | 38.64 |  | A | -0.0627 | 0.0195 |
| rs1832007 | 10 | 5,254,847 | A | 0.0327 | 0.0047 | 177,504 | 2.73E-4 | 48.41 |  | A | -0.0328 | 0.0249 |
| rs10761762 | 10 | 65,184,717 | T | 0.0270 | 0.0033 | 177,823 | 3.76E-4 | 66.94 |  | T | 0.0155 | 0.0176 |
| rs2068888 | 10 | 94,839,642 | G | 0.0241 | 0.0034 | 177,712 | 2.83E-4 | 50.24 |  | G | -0.0198 | 0.0177 |
| rs11820504 | 11 | 116,529,442 | C | 0.0604 | 0.0044 | 172,813 | 1.09E-3 | 188.44 |  | C | -0.0495 | 0.0238 |
| rs10790162 | 11 | 116,639,104 | A | 0.2305 | 0.0065 | 177,771 | 7.02E-3 | 1257.51 |  | A | 0.0639 | 0.0348 |
| rs10501321 | 11 | 47,294,626 | T | 0.0216 | 0.0035 | 177,680 | 2.14E-4 | 38.09 |  | T | -0.0189 | 0.0191 |
| rs174535 | 11 | 61,551,356 | C | 0.0470 | 0.0034 | 177,773 | 1.07E-3 | 191.09 |  | C | 0.0175 | 0.0187 |
| rs11057408 | 12 | 124,464,836 | G | 0.0258 | 0.0035 | 174,454 | 3.11E-4 | 54.34 |  | G | 0.0006 | 0.0186 |
| rs1321257 | 1 | 230,305,312 | G | 0.0402 | 0.0034 | 177,758 | 7.86E-4 | 139.79 |  | G | 0.0002 | 0.0181 |
| rs11613352 | 12 | 57,792,580 | C | 0.0280 | 0.0039 | 177,799 | 2.90E-4 | 51.54 |  | C | -0.0382 | 0.0213 |
| rs12748152 | 1 | 27,138,393 | T | 0.0372 | 0.0059 | 177,762 | 2.24E-4 | 39.75 |  | T | -0.0064 | 0.0331 |
| rs17513135 | 1 | 40,035,686 | T | 0.0220 | 0.0039 | 174,742 | 1.82E-4 | 31.82 |  | T | 0.0385 | 0.0208 |
| rs16948098 | 15 | 44,219,607 | A | 0.0800 | 0.0089 | 163,328 | 4.94E-4 | 80.80 |  | A | -0.0191 | 0.0433 |
| rs2043085 | 15 | 58,680,954 | T | 0.0327 | 0.0034 | 176,147 | 5.25E-4 | 92.50 |  | T | -0.0220 | 0.0183 |
| rs588136 | 15 | 58,730,498 | C | 0.0495 | 0.0041 | 176,173 | 8.27E-4 | 145.76 |  | C | 0.0164 | 0.0222 |
| rs3198697 | 16 | 15,129,940 | C | 0.0198 | 0.0034 | 175,934 | 1.93E-4 | 33.91 |  | C | 0.0071 | 0.018 |
| rs4587594 | 1 | 63,133,930 | G | 0.0694 | 0.0035 | 177,772 | 2.21E-3 | 393.17 |  | G | -0.0030 | 0.0188 |
| rs749671 | 16 | 31,088,347 | G | 0.0211 | 0.0034 | 176,205 | 2.19E-4 | 38.51 |  | G | 0.0035 | 0.0183 |
| rs1800775 | 16 | 56,995,236 | C | 0.0396 | 0.0035 | 172,715 | 7.41E-4 | 128.01 |  | C | -0.0103 | 0.0178 |
| rs8077889 | 17 | 41,878,166 | C | 0.0252 | 0.0042 | 176,194 | 2.04E-4 | 36.00 |  | C | -0.0169 | 0.0217 |
| rs10401969 | 19 | 19,407,718 | T | 0.1210 | 0.0065 | 176,173 | 1.96E-3 | 346.53 |  | T | -0.0075 | 0.0345 |
| rs731839 | 19 | 33,899,065 | G | 0.0224 | 0.0036 | 176,161 | 2.20E-4 | 38.72 |  | G | 0.0257 | 0.0188 |
| rs439401 | 19 | 45,414,451 | C | 0.0659 | 0.0038 | 152,584 | 1.97E-3 | 300.74 |  | C | 0.0008 | 0.0185 |
| rs3760627 | 19 | 45,457,180 | C | 0.0189 | 0.0034 | 176,201 | 1.75E-4 | 30.90 |  | C | 0.0073 | 0.0178 |
| rs7248104 | 19 | 7,224,431 | G | 0.0222 | 0.0034 | 176,083 | 2.42E-4 | 42.63 |  | G | 0.0012 | 0.0179 |
| rs6029143 | 20 | 39,118,662 | C | 0.0388 | 0.0071 | 176,257 | 1.69E-4 | 29.86 |  | C | 0.0510 | 0.036 |
| rs4810479 | 20 | 44,545,048 | C | 0.0474 | 0.0038 | 176,192 | 8.82E-4 | 155.59 |  | C | -0.0174 | 0.0202 |
| rs13389219 | 2 | 165,528,876 | C | 0.0271 | 0.0034 | 177,783 | 3.57E-4 | 63.53 |  | C | 0.0165 | 0.0181 |
| rs676210 | 2 | 21,231,524 | G | 0.0733 | 0.0039 | 177,782 | 1.98E-3 | 353.24 |  | G | 0.0471 | 0.0213 |
| rs2972146 | 2 | 227,100,698 | T | 0.0281 | 0.0034 | 174,704 | 3.91E-4 | 68.30 |  | T | -0.0319 | 0.0183 |
| rs3761445 | 22 | 38,595,411 | A | 0.0232 | 0.0034 | 175,846 | 2.65E-4 | 46.56 |  | A | 0.0358 | 0.018 |
| rs1260326 | 2 | 27,730,940 | T | 0.1148 | 0.0034 | 177,765 | 6.37E-3 | 1140.04 |  | T | 0.0164 | 0.0179 |
| rs10440120 | 3 | 12,486,964 | C | 0.0306 | 0.0044 | 174,886 | 2.76E-4 | 48.37 |  | C | -0.0148 | 0.024 |
| rs645040 | 3 | 135,926,622 | T | 0.0293 | 0.004 | 177,779 | 3.02E-4 | 53.66 |  | T | -0.0225 | 0.0213 |
| rs6831256 | 4 | 3,473,139 | G | 0.0258 | 0.0035 | 177,495 | 3.06E-4 | 54.34 |  | G | -0.0113 | 0.0179 |
| rs442177 | 4 | 88,030,261 | T | 0.0309 | 0.0033 | 177,798 | 4.93E-4 | 87.68 |  | T | 0.0119 | 0.018 |
| rs6882076 | 5 | 156,390,297 | C | 0.0286 | 0.0035 | 177,778 | 3.75E-4 | 66.77 |  | C | 0.0062 | 0.0183 |
| rs9686661 | 5 | 55,861,786 | T | 0.0379 | 0.0044 | 177,050 | 4.19E-4 | 74.19 |  | T | 0.0124 | 0.0226 |
| rs719726 | 6 | 127,414,801 | T | 0.0199 | 0.0035 | 168,319 | 1.92E-4 | 32.33 |  | T | -0.0311 | 0.018 |
| rs634869 | 6 | 139,831,757 | T | 0.0272 | 0.0033 | 177,755 | 3.82E-4 | 67.94 |  | T | -0.0025 | 0.0179 |
| rs2665357 | 6 | 160,848,167 | C | 0.0212 | 0.0033 | 172,850 | 2.39E-4 | 41.27 |  | C | 0.0210 | 0.0177 |
| rs2239520 | 6 | 31,088,922 | G | 0.0236 | 0.0037 | 151,047 | 2.69E-4 | 40.68 |  | G | -0.0193 | 0.0179 |
| rs2247056 | 6 | 31,265,490 | C | 0.0378 | 0.0039 | 174,062 | 5.39E-4 | 93.94 |  | C | -0.0250 | 0.0206 |
| rs998584 | 6 | 43,757,896 | A | 0.0293 | 0.0037 | 174,573 | 3.59E-4 | 62.71 |  | A | 0.0011 | 0.0187 |
| rs38855 | 7 | 116,358,044 | A | 0.0187 | 0.0033 | 177,825 | 1.81E-4 | 32.11 |  | A | 0.0008 | 0.0177 |
| rs287621 | 7 | 130,435,181 | T | 0.0222 | 0.0037 | 177,813 | 2.02E-4 | 36.00 |  | T | 0.0154 | 0.0195 |
| rs4719841 | 7 | 25,997,536 | G | 0.0232 | 0.0034 | 177,775 | 2.62E-4 | 46.56 |  | G | 0.0024 | 0.018 |
| rs11974409 | 7 | 72,989,390 | A | 0.0899 | 0.0042 | 177,786 | 2.57E-3 | 458.16 |  | A | -0.0136 | 0.0229 |
| rs6995541 | 8 | 10,671,260 | G | 0.0265 | 0.0037 | 177,486 | 2.89E-4 | 51.30 |  | G | -0.0007 | 0.0196 |
| rs2954022 | 8 | 126,482,621 | C | 0.0780 | 0.0033 | 177,750 | 3.13E-3 | 558.67 |  | C | -0.0027 | 0.0176 |
| rs12676857 | 8 | 18,266,572 | C | 0.0332 | 0.0046 | 177,732 | 2.93E-4 | 52.09 |  | C | 0.0140 | 0.0247 |
| rs12678919 | 8 | 19,844,222 | A | 0.1702 | 0.0056 | 177,750 | 5.17E-3 | 923.72 |  | A | -0.0301 | 0.0291 |
| rs4738684 | 8 | 59,393,273 | A | 0.0205 | 0.0035 | 177,790 | 1.93E-4 | 34.31 |  | A | -0.0170 | 0.0186 |

In the Allele column, the first one is the effect allele. TG: triglycerides, ALS: amyotrophic lateral sclerosis, Chr: chromosome, SNP: single-nucleotide polymorphism, Beta: SNP effect size, SE: standard error; *N* and *F* are respectively the sample size and *F* statistic for each SNP. These instruments collectively explain 4.92% HDL variation (i.e. proportion of variance explained [PVE] by SNPs = 4.92%), we compute the *F* statistic for each instrument via $F_{i} = \text{PVE}_{i}\text{(}N_{i} -1-k\text{)/}(k-k\times\text{PVE}_{i})$, where *N_i_* is the sample size for instrument *i* and *k* is the number of total instruments. All the *F* statistics are larger than 10 (ranging from 29.86 to 1257.51). The overall *F* statistic for the 54 instrumental variables is 167.92 (with PVE = 4.92%, *k* = 54 and *N* = 175,286), suggesting that the selected genetic variants have sufficient strong effects for instrument and the bias of weak instrument is unlikely.

**Table S6.** Index SNPs used as instruments for HDL and their effects, standard errors for ASL in the East Asian population

| SNP | CHR | Position (GRCh37) | HDL | | | | | |  | ALS | | |
| --- | --- | --- | --- | --- | --- | --- | --- | --- | --- | --- | --- | --- |
|  |  |  | Allele | Beta | SE | *p* | PVE | *F* |  | Allele | Beta | SE |
| rs3741297 | 11 | 116,657,667 | C | -0.2579 | 0.0097 | 2.52E-157 | 1.00E-2 | 714.23 |  | C | -0.0341 | 0.0229 |
| rs79105258 | 12 | 111,718,231 | C | -0.1239 | 0.0065 | 1.69E-80 | 5.08E-3 | 360.77 |  | C | 0.0028 | 0.0138 |
| rs1883025 | 9 | 107,664,301 | C | -0.1081 | 0.0059 | 3.03E-76 | 4.81E-3 | 341.45 |  | C | 0.0169 | 0.0125 |
| rs429358 | 19 | 45,411,941 | T | -0.0895 | 0.0088 | 2.43E-24 | 1.46E-3 | 103.62 |  | T | 0.0043 | 0.0172 |
| rs11067592 | 12 | 110,069,190 | G | -0.0886 | 0.0102 | 5.12E-18 | 1.06E-3 | 74.78 |  | G | 0.0181 | 0.0238 |
| rs76083992 | 20 | 44,544,798 | C | -0.0851 | 0.0112 | 3.53E-14 | 8.12E-4 | 57.40 |  | C | -0.0165 | 0.0212 |
| rs11236520 | 11 | 75,460,669 | G | -0.0414 | 0.0072 | 7.97E-9 | 4.71E-4 | 33.28 |  | G | -0.0309 | 0.0162 |
| rs7445 | 22 | 21,977,047 | C | -0.0409 | 0.0053 | 1.25E-14 | 8.41E-4 | 59.46 |  | C | -0.0017 | 0.0102 |
| rs4917630 | 10 | 114,019,830 | G | -0.0389 | 0.0054 | 7.56E-13 | 7.27E-4 | 51.39 |  | G | 0.0079 | 0.0105 |
| rs6685271 | 1 | 93,634,590 | C | -0.0387 | 0.0060 | 1.37E-10 | 5.83E-4 | 41.22 |  | C | 0.0032 | 0.0106 |
| rs28577594 | 12 | 123,895,906 | G | -0.0366 | 0.0061 | 1.67E-9 | 5.14E-4 | 36.33 |  | G | 0.0120 | 0.0110 |
| rs2257129 | 10 | 122,898,697 | T | -0.0365 | 0.0057 | 1.85E-10 | 5.75E-4 | 40.62 |  | T | -0.0116 | 0.0103 |
| rs117350179 | 3 | 12,374,332 | C | -0.0343 | 0.0062 | 2.96E-8 | 4.35E-4 | 30.73 |  | C | 0.0081 | 0.0114 |
| rs235314 | 21 | 46,271,452 | C | -0.0332 | 0.0053 | 3.30E-10 | 5.58E-4 | 39.48 |  | C | -0.0013 | 0.0102 |
| rs7847628 | 9 | 123,631,225 | A | -0.0320 | 0.0057 | 1.62E-8 | 4.51E-4 | 31.90 |  | A | -0.0139 | 0.0102 |
| rs1358980 | 6 | 43,764,551 | C | -0.0313 | 0.0053 | 2.69E-9 | 5.01E-4 | 35.39 |  | C | -0.0108 | 0.0101 |
| rs1026422 | 7 | 50,319,807 | G | 0.0327 | 0.0052 | 4.84E-10 | 5.48E-4 | 38.74 |  | G | -0.0107 | 0.0115 |
| rs7895716 | 10 | 94,783,777 | C | 0.0353 | 0.0055 | 1.24E-10 | 5.85E-4 | 41.39 |  | C | 0.0019 | 0.0127 |
| rs2144300 | 1 | 230,294,916 | C | 0.0366 | 0.0067 | 4.25E-8 | 4.25E-4 | 30.02 |  | C | 0.0142 | 0.0133 |
| rs28366301 | 6 | 32,560,883 | G | 0.0369 | 0.0057 | 7.58E-11 | 5.99E-4 | 42.37 |  | G | -0.0052 | 0.0110 |
| rs2925979 | 16 | 81,534,790 | T | 0.0397 | 0.0055 | 7.40E-13 | 7.27E-4 | 51.43 |  | T | -0.0193 | 0.0104 |
| rs76213020 | 14 | 52,436,005 | A | 0.0452 | 0.0079 | 1.19E-8 | 4.60E-4 | 32.50 |  | A | -0.0148 | 0.0146 |
| rs3808447 | 8 | 116,575,459 | G | 0.0485 | 0.0062 | 3.64E-15 | 8.75E-4 | 61.88 |  | G | -0.0276 | 0.0129 |
| rs7778167 | 7 | 127,851,628 | G | 0.0556 | 0.0083 | 1.99E-11 | 6.36E-4 | 44.97 |  | G | -0.0064 | 0.0173 |
| rs56303487 | 16 | 68,029,739 | C | 0.0692 | 0.0077 | 3.09E-19 | 1.14E-3 | 80.39 |  | C | -0.0077 | 0.0153 |
| rs67053123 | 12 | 125,353,810 | T | 0.0706 | 0.0059 | 1.38E-32 | 2.00E-3 | 141.33 |  | T | -0.0087 | 0.0121 |
| rs11082764 | 18 | 47,119,579 | A | 0.0784 | 0.0052 | 5.63E-51 | 3.18E-3 | 225.54 |  | A | -0.0105 | 0.0103 |
| rs2070895 | 15 | 58,723,939 | G | 0.1322 | 0.0052 | 1.13E-140 | 8.94E-3 | 637.70 |  | G | -0.0039 | 0.0105 |
| rs189213544 | 7 | 80,342,775 | G | 0.1447 | 0.0154 | 4.88E-21 | 1.25E-3 | 88.63 |  | NA | NA | NA |
| rs325 | 8 | 19,819,328 | T | 0.1483 | 0.0079 | 2.09E-79 | 5.02E-3 | 356.16 |  | T | -0.0108 | 0.0187 |
| rs56156922 | 16 | 56,987,369 | T | 0.2656 | 0.0064 | 2.18E-371 | 2.35E-2 | 1698.76 |  | T | 0.0010 | 0.0138 |

In the Allele column, only the effect allele is presented. HDL: high density lipoprotein, ALS: amyotrophic lateral sclerosis, Chr: chromosome, SNP: single-nucleotide polymorphism, Beta: SNP effect size, SE: standard error. The estimated proportion of variance explained (PVE) by the selected SNPs are 7.75% for HDL. We compute the *F* statistic for each instrument via $F_{i} = \text{PVE}_{i}\text{(}N_{i} -1-k\text{)/}(k-k\times\text{PVE}_{i})$. All the *F* statistics are larger than 10 (ranging from 30.02 to 1698.76). The overall *F* statistic is 197.78 for HDL with PVE = 7.75%, *N* = 70,657 and *k* = 30.

**Table S7.** Index SNPs used as instruments for LDL and the effects, standard errors for ASL in the East Asian population

| SNP | CHR | Position (GRCh37) | LDL | | | | | |  | ALS | | |
| --- | --- | --- | --- | --- | --- | --- | --- | --- | --- | --- | --- | --- |
|  |  |  | Allele | Beta | SE | *p* | PVE | *F* |  | Allele | Beta | SE |
| rs769446 | 19 | 45,408,628 | T | -0.5141 | 0.0134 | 2.96e-322 | 1.98E-2 | 1471.88 |  | T | 0.0031 | 0.0188 |
| rs77303550 | 16 | 72,079,657 | C | -0.0564 | 0.0070 | 6.85E-16 | 8.94E-4 | 65.17 |  | C | -0.0290 | 0.0119 |
| rs113932726 | 11 | 116,650,638 | C | -0.0546 | 0.0097 | 1.70E-8 | 4.36E-4 | 31.82 |  | C | -0.0341 | 0.0229 |
| rs41280378 | 10 | 74,692,646 | T | -0.0485 | 0.0063 | 9.19E-15 | 8.24E-4 | 60.05 |  | T | -0.0160 | 0.0112 |
| rs3093679 | 17 | 26,664,215 | G | -0.0428 | 0.0071 | 1.92E-9 | 4.95E-4 | 36.05 |  | G | -0.0071 | 0.0122 |
| rs28537499 | 7 | 26,015,392 | A | -0.0339 | 0.0053 | 1.84E-10 | 5.57E-4 | 40.62 |  | A | 0.0065 | 0.0103 |
| rs75214121 | 8 | 59,398,276 | C | -0.0333 | 0.0058 | 8.88E-9 | 4.54E-4 | 33.07 |  | C | -0.0099 | 0.0123 |
| rs2001846 | 8 | 126,478,450 | T | -0.0305 | 0.0052 | 6.34E-9 | 4.63E-4 | 33.73 |  | T | -0.0080 | 0.0102 |
| rs7315593 | 12 | 113,277,970 | G | -0.0302 | 0.0052 | 8.45E-9 | 4.55E-4 | 33.16 |  | G | 0.0019 | 0.0103 |
| rs56903760 | 15 | 58,692,681 | C | 0.0334 | 0.0059 | 1.65E-8 | 4.37E-4 | 31.86 |  | C | -0.0078 | 0.0110 |
| rs56668103 | 20 | 39,826,079 | T | 0.0338 | 0.0059 | 7.28E-9 | 4.59E-4 | 33.46 |  | T | 0.0000 | 0.0117 |
| rs75061399 | 12 | 100,942,077 | G | 0.0340 | 0.0062 | 4.80E-8 | 4.09E-4 | 29.80 |  | G | -0.0108 | 0.0112 |
| rs553427 | 1 | 234,852,760 | C | 0.0348 | 0.0058 | 1.46E-9 | 5.02E-4 | 36.58 |  | C | 0.0032 | 0.0116 |
| rs6882076 | 5 | 156,390,297 | T | 0.0428 | 0.0075 | 1.05E-8 | 4.49E-4 | 32.74 |  | T | 0.0068 | 0.0117 |
| rs17145738 | 7 | 72,982,874 | C | 0.0500 | 0.0085 | 4.83E-9 | 4.70E-4 | 34.27 |  | C | 0.0280 | 0.0153 |
| rs9411378 | 9 | 136,145,425 | C | 0.0562 | 0.0062 | 1.16E-19 | 1.13E-3 | 82.31 |  | C | -0.0114 | 0.0125 |
| rs79105258 | 12 | 111,718,231 | C | 0.0598 | 0.0065 | 2.84E-20 | 1.17E-3 | 85.08 |  | C | 0.0028 | 0.0138 |
| rs3846661 | 5 | 74,639,178 | A | 0.0632 | 0.0055 | 4.70E-31 | 1.84E-3 | 134.25 |  | A | 0.0205 | 0.0100 |
| rs2738464 | 19 | 11,242,307 | G | 0.0746 | 0.0056 | 3.84E-40 | 2.41E-3 | 175.85 |  | G | 0.0110 | 0.0112 |
| rs660240 | 1 | 109,817,838 | T | 0.1122 | 0.0101 | 1.79E-28 | 1.68E-3 | 122.67 |  | T | 0.0330 | 0.0210 |
| rs75352129 | 2 | 20,848,192 | C | 0.2101 | 0.0130 | 5.64E-59 | 3.58E-3 | 261.99 |  | C | 0.1020 | 0.0353 |
| rs147943615 | 1 | 56,368,037 | A | 0.2224 | 0.0204 | 1.33E-27 | 1.62E-3 | 118.50 |  | NA | NA | NA |

In the Allele column, only the effect allele is presented. LDL: low density lipoprotein, ALS: amyotrophic lateral sclerosis, Chr: chromosome, SNP: single-nucleotide polymorphism, Beta: SNP effect size, SE: standard error. The estimated proportion of variance explained (PVE) by the selected SNPs are 3.89% for LDL. We compute the *F* statistic for each instrument via $F_{i} = \text{PVE}_{i}\text{(}N_{i} -1-k\text{)/}(k-k\times\text{PVE}_{i})$, where *N_i_* is the sample size for instrument *i* and *k* is the number of total instruments. All the *F* statistics are larger than 10 (ranging from 29.80 to 1471.88). The overall *F* statistic is 140.40 for LDL with PVE = 3.89%, *N* = 72,866 and *k* = 21.

**Table S8.** Index SNPs used as instruments for TC and the effects, standard errors for ASL in the East Asian population

| SNP | CHR | Position (GRCh37) | TC | | | | | |  | ALS | | |
| --- | --- | --- | --- | --- | --- | --- | --- | --- | --- | --- | --- | --- |
|  |  |  | Allele | Beta | SE | *p* | PVE | *F* |  | Allele | Beta | SE |
| rs769446 | 19 | 45,408,628 | T | -0.2845 | 0.0098 | 5.86E-187 | 6.58E-3 | 850.21 |  | T | 0.0031 | 0.0188 |
| rs75542613 | 11 | 116,679,155 | G | -0.0686 | 0.0087 | 3.40E-15 | 4.83E-4 | 62.00 |  | G | 0.0059 | 0.0218 |
| rs45576433 | 1 | 55,517,301 | A | -0.0619 | 0.0062 | 1.46E-23 | 7.79E-4 | 100.06 |  | A | -0.0245 | 0.0163 |
| rs77303550 | 16 | 72,079,657 | C | -0.0607 | 0.0052 | 2.64E-31 | 1.05E-3 | 135.45 |  | C | -0.0290 | 0.0119 |
| rs2575876 | 9 | 107,665,739 | G | -0.0562 | 0.0044 | 3.83E-38 | 1.30E-3 | 166.72 |  | G | 0.0116 | 0.0126 |
| rs78295286 | 10 | 74,665,524 | G | -0.0458 | 0.0077 | 2.64E-9 | 2.76E-4 | 35.43 |  | NA | NA | NA |
| rs116961202 | 8 | 59,382,311 | A | -0.0377 | 0.0044 | 4.63E-18 | 5.84E-4 | 75.01 |  | A | -0.0088 | 0.0123 |
| rs34010237 | 19 | 50,012,574 | G | -0.0367 | 0.0059 | 6.40E-10 | 2.98E-4 | 38.19 |  | G | 0.0211 | 0.0129 |
| rs1260326 | 2 | 27,730,940 | T | -0.0353 | 0.0039 | 2.80E-19 | 6.28E-4 | 80.57 |  | T | -0.0011 | 0.0101 |
| rs61775910 | 1 | 62,993,403 | G | -0.0352 | 0.0056 | 3.72E-10 | 3.06E-4 | 39.25 |  | G | 0.0182 | 0.0127 |
| rs6982502 | 8 | 126,479,362 | C | -0.0321 | 0.0039 | 1.95E-16 | 5.27E-4 | 67.65 |  | C | -0.0090 | 0.0102 |
| rs57594838 | 10 | 114,025,182 | T | -0.0268 | 0.0043 | 5.94E-10 | 2.99E-4 | 38.33 |  | T | 0.0038 | 0.0108 |
| rs2737252 | 8 | 116,663,898 | G | -0.0266 | 0.0044 | 1.43E-9 | 2.85E-4 | 36.63 |  | G | 0.0051 | 0.0111 |
| rs62074014 | 17 | 45,668,509 | A | -0.0265 | 0.0045 | 2.99E-9 | 2.74E-4 | 35.19 |  | A | -0.0060 | 0.0104 |
| rs12713468 | 2 | 62,983,213 | G | -0.0255 | 0.0047 | 4.68E-8 | 2.33E-4 | 29.85 |  | G | 0.0013 | 0.0108 |
| rs2243616 | 12 | 121,406,370 | G | -0.0250 | 0.0040 | 5.50E-10 | 3.00E-4 | 38.48 |  | G | -0.0146 | 0.0103 |
| rs6970673 | 7 | 26,014,642 | C | -0.0227 | 0.0040 | 1.12E-8 | 2.54E-4 | 32.61 |  | C | 0.0063 | 0.0103 |
| rs58143210 | 4 | 3,425,030 | C | -0.0224 | 0.0040 | 2.83E-8 | 2.40E-4 | 30.83 |  | C | 0.0138 | 0.0099 |
| rs55935382 | 7 | 50,289,669 | C | 0.0227 | 0.0039 | 7.73E-9 | 2.60E-4 | 33.34 |  | C | -0.0066 | 0.0117 |
| rs67053123 | 12 | 125,353,810 | T | 0.0257 | 0.0044 | 6.18E-9 | 2.63E-4 | 33.79 |  | T | -0.0087 | 0.0121 |
| rs56668103 | 20 | 39,826,079 | T | 0.0257 | 0.0044 | 3.91E-9 | 2.70E-4 | 34.66 |  | T | 0.0000 | 0.0117 |
| rs557933 | 1 | 234,853,268 | A | 0.0290 | 0.0043 | 1.96E-11 | 3.51E-4 | 45.01 |  | A | 0.0035 | 0.0116 |
| rs97384 | 11 | 61,624,181 | T | 0.0364 | 0.0044 | 1.06E-16 | 5.36E-4 | 68.86 |  | T | 0.0097 | 0.0105 |
| rs3786247 | 18 | 47,118,923 | T | 0.0443 | 0.0039 | 5.27E-30 | 1.01E-3 | 129.51 |  | T | -0.0110 | 0.0103 |
| rs11745603 | 5 | 156,394,518 | C | 0.0452 | 0.0055 | 3.17E-16 | 5.20E-4 | 66.71 |  | C | 0.0063 | 0.0117 |
| rs11748027 | 5 | 74,909,972 | C | 0.0512 | 0.0041 | 3.49E-36 | 1.23E-3 | 157.71 |  | C | 0.0209 | 0.0100 |
| rs9411378 | 9 | 136,145,425 | C | 0.0512 | 0.0046 | 2.77E-28 | 9.47E-4 | 121.63 |  | C | -0.0114 | 0.0125 |
| rs56156922 | 16 | 56,987,369 | T | 0.0570 | 0.0048 | 2.31E-32 | 1.09E-3 | 140.33 |  | T | 0.0010 | 0.0138 |
| rs1800588 | 15 | 58,723,675 | C | 0.0597 | 0.0039 | 4.25E-53 | 1.83E-3 | 235.25 |  | C | 0.0023 | 0.0105 |
| rs2738464 | 19 | 11,242,307 | G | 0.0616 | 0.0042 | 8.48E-49 | 1.68E-3 | 215.58 |  | G | 0.0110 | 0.0112 |
| rs646776 | 1 | 109,818,530 | C | 0.0816 | 0.0076 | 4.53E-27 | 9.04E-4 | 116.10 |  | C | 0.0253 | 0.0199 |
| rs75352129 | 2 | 20,848,192 | C | 0.1304 | 0.0099 | 2.01E-39 | 1.34E-3 | 172.48 |  | C | 0.1020 | 0.0353 |

In the Allele column, only the effect allele is presented. TC: total cholesterol, ALS: amyotrophic lateral sclerosis, Chr: chromosome, SNP: single-nucleotide polymorphism, Beta: SNP effect size, SE: standard error. The estimated proportion of variance explained (PVE) by the selected SNPs are 2.67% for TC. We compute the *F* statistic for each instrument via $F_{i} = \text{PVE}_{i}\text{(}N_{i} -1-k\text{)/}(k-k\times\text{PVE}_{i})$, where *N_i_* is the sample size for instrument *i* and *k* is the number of total instruments. All the *F* statistics are larger than 10 (ranging from 29.85 to 850.21). The overall *F* statistic is 113.51 for TC with PVE = 2.67%, *N* = 128,305 and *k* = 31.

**Table S9.** Index SNPs used as instruments for TG and the effects, standard errors for ASL in the East Asian population

| SNP | CHR | Position (GRCh37) | TG | | | | | |  | ALS | | |
| --- | --- | --- | --- | --- | --- | --- | --- | --- | --- | --- | --- | --- |
|  |  |  | Allele | Beta | SE | *p* | PVE | *F* |  | Allele | Beta | SE |
| rs651821 | 11 | 116,662,579 | C | -0.2208 | 0.0044 | 1.07E-537 | 2.28E-2 | 2464.11 |  | C | 0.0025 | 0.0117 |
| rs1059611 | 8 | 19,824,563 | T | -0.1698 | 0.0064 | 1.49E-154 | 6.60E-3 | 701.04 |  | T | -0.0033 | 0.0184 |
| rs12531645 | 7 | 73,023,881 | G | -0.1056 | 0.0072 | 3.23E-49 | 2.06E-3 | 217.46 |  | G | 0.0286 | 0.0154 |
| rs1260326 | 2 | 27,730,940 | T | -0.0888 | 0.0043 | 1.69E-94 | 4.01E-3 | 425.37 |  | T | -0.0011 | 0.0101 |
| rs35529421 | 1 | 62,965,621 | T | -0.0748 | 0.0063 | 3.57E-32 | 1.32E-3 | 139.40 |  | T | 0.0203 | 0.0128 |
| rs58542926 | 19 | 19,379,549 | C | -0.0638 | 0.0086 | 9.10E-14 | 5.26E-4 | 55.54 |  | C | 0.0098 | 0.0208 |
| rs2954021 | 8 | 126,482,077 | A | -0.0578 | 0.0043 | 7.43E-42 | 1.74E-3 | 183.78 |  | A | -0.0091 | 0.0102 |
| rs75766425 | 14 | 52,511,911 | G | -0.0379 | 0.0067 | 1.27E-8 | 3.07E-4 | 32.38 |  | G | -0.0269 | 0.0152 |
| rs56156922 | 16 | 56,987,369 | T | -0.0373 | 0.0053 | 1.41E-12 | 4.75E-4 | 50.18 |  | T | 0.0010 | 0.0138 |
| rs4411227 | 10 | 94,831,513 | C | -0.0369 | 0.0045 | 3.57E-16 | 6.29E-4 | 66.47 |  | C | 0.0106 | 0.0131 |
| rs2278426 | 19 | 11,350,488 | C | -0.0357 | 0.0048 | 1.62E-13 | 5.15E-4 | 54.41 |  | C | 0.0183 | 0.0114 |
| rs3752442 | 4 | 3,446,883 | A | -0.0335 | 0.0043 | 1.27E-14 | 5.63E-4 | 59.44 |  | A | 0.0041 | 0.0100 |
| rs7916868 | 10 | 64,988,931 | A | -0.0329 | 0.0051 | 1.41E-10 | 3.90E-4 | 41.16 |  | A | -0.0026 | 0.0102 |
| rs2144300 | 1 | 230,294,916 | C | -0.0299 | 0.0054 | 4.23E-8 | 2.84E-4 | 30.03 |  | C | 0.0142 | 0.0133 |
| rs2679617 | 15 | 70,207,077 | A | -0.0271 | 0.0049 | 2.38E-8 | 2.95E-4 | 31.16 |  | A | -0.0027 | 0.0106 |
| rs1495743 | 8 | 18,273,300 | G | -0.0261 | 0.0046 | 1.30E-8 | 3.06E-4 | 32.32 |  | G | 0.0037 | 0.0101 |
| rs154254 | 5 | 55,820,584 | G | 0.0253 | 0.0044 | 7.59E-9 | 3.16E-4 | 33.38 |  | G | 0.0103 | 0.0102 |
| rs4389834 | 7 | 25,987,129 | G | 0.0261 | 0.0044 | 2.28E-9 | 3.38E-4 | 35.71 |  | G | -0.0002 | 0.0101 |
| rs2114273 | 1 | 93,854,517 | T | 0.0281 | 0.0045 | 5.95E-10 | 3.63E-4 | 38.33 |  | T | 0.0096 | 0.0106 |
| rs174551 | 11 | 61,573,684 | T | 0.0352 | 0.0045 | 8.15E-15 | 5.71E-4 | 60.29 |  | T | -0.0047 | 0.0107 |
| rs35469118 | 4 | 88,168,178 | C | 0.0353 | 0.0045 | 9.04E-15 | 5.69E-4 | 60.07 |  | C | -0.0073 | 0.0108 |
| rs2146324 | 6 | 43,756,863 | A | 0.0486 | 0.0075 | 1.19E-10 | 3.93E-4 | 41.48 |  | A | -0.0071 | 0.0131 |
| rs12992267 | 2 | 21,215,645 | C | 0.0510 | 0.0076 | 1.46E-11 | 4.31E-4 | 45.58 |  | C | 0.0220 | 0.0186 |
| rs1800588 | 15 | 58,723,675 | C | 0.0640 | 0.0043 | 6.22E-51 | 2.13E-3 | 225.31 |  | C | 0.0023 | 0.0105 |
| rs76083992 | 20 | 44,544,798 | C | 0.0704 | 0.0091 | 1.33E-14 | 5.62E-4 | 59.34 |  | C | -0.0165 | 0.0212 |
| rs75627662 | 19 | 45,413,576 | C | 0.0907 | 0.0067 | 5.65E-42 | 1.74E-3 | 184.28 |  | C | 0.0068 | 0.0133 |

In the Allele column, only the effect allele is presented. TG: triglycerides, ALS: amyotrophic lateral sclerosis, Chr: chromosome, SNP: single-nucleotide polymorphism, Beta: SNP effect size, SE: standard error. The estimated proportion of variance explained (PVE) by the selected SNPs are 5.02% for TG. We compute the *F* statistic for each instrument via $F_{i} = \text{PVE}_{i}\text{(}N_{i} -1-k\text{)/}(k-k\times\text{PVE}_{i})$, where *N_i_* is the sample size for instrument *i* and *k* is the number of total instruments. All the *F* statistics are larger than 10 (ranging from 30.03 to 2464.11). The overall *F* statistic is 214.60 for TG with PVE = 5.02%, *N* = 105,597 and *k* = 26.

**Table S10.** Causal effect estimates of LDL after removing other potential confounding lipid traits

| Methods | lipids | *k* | European | | | | *k* |  | East Asian | | | |
| --- | --- | --- | --- | --- | --- | --- | --- | --- | --- | --- | --- | --- |
|  |  |  | Beat/OR | SE | *z* value | *p* value |  |  | Beat/OR | SE | *z* value | *p* value |
| Instruments-specific  MR | HDL | 73 | 0.03/1.03 | 0.06 | -0.64 | 5.25E-1 | 25 |  | -0.05/0.96 | 0.04 | -1.21 | 2.26E-1 |
|  | **LDL** | **38** | **0.14/1.14** | **0.06** | **2.00** | **4.50E-2** | **14** |  | **0.19/1.21** | **0.07** | **2.81** | **5.04E-3** |
|  | TC | 40 | 0.08/1.09 | 0.06 | 1.77 | 7.76E-2 | 21 |  | 0.06/1.06 | 0.08 | 0.73 | 4.63E-1 |
|  | TG | 46 | -0.01/0.99 | 0.05 | -0.01 | 9.90E-1 | 21 |  | -0.03/0.97 | 0.04 | -0.68 | 4.98E-1 |
| Residual-based  MR regression | HDL |  | **0.15/1.16** | 0.07 | 2.21 | 2.70E-2 |  |  | **0.20/1.22** | 0.05 | 3.90 | 9.78E-5 |
|  | TG |  | **0.13/1.14** | 0.07 | 1.94 | 5.28E-2 |  |  | **0.17/1.18** | 0.06 | 2.78 | 5.44E-3 |
|  | HDL + TG |  | **0.15/1.16** | 0.07 | 2.21 | 2.73E-2 |  |  | **0.18/1.20** | 0.05 | 3.41 | 6.45E-4 |
| Joint MR regression | LDL |  | **0.19/1.20** | 0.08 | 2.48 | 1.33E-2 |  |  | **0.20/1.22** | 0.06 | 3.43 | 6.15E-4 |

In both residual-based and joint MR regressions TC was not considered due to high correlation with LDL (i.e. *r* = 0.992 and 0.787 in the European and East Asian populations, respectively). The threshold of lipid-specific instruments was set to 5.00E-8. Here, *k* is the number of instruments used in the analysis. MR: Mendelian randomization.

**Table S11.** Baseline Characteristics of the Chinese lipids GWAS

| Studies | Sample size | Male/female | age | BMI (kg/m^2^) | HDL (mg/dL) | LDL(mg/dL) | TC (mg/dL) | TG (mg/dL) |
| --- | --- | --- | --- | --- | --- | --- | --- | --- |
| CAS | 3,998 | 1,870/2,128 | 52.53 (7.69) | 25.18 (3.79) | 49.92 (12.42) | 105.58 (30.35) | 185.84 (35.79) | 159.24 (115.52) |
| BAS | 466 | 363/103 | 51.07 (9.53) | 24.64 (3.36) | 49.78 (12.38) | 119.79 (32.94) | 195.34 (37.05) | 128.04 (98.84) |
| GenSalt | 1,881 | 993/888 | 38.72 (9.53) | 23.35 (3.18) | 50.97 (11.31) | 95.25 (27.30) | 170.70 (33.65) | 123.47 (78.20) |
| FAMHES | 1,999 | 1,999/0 | 37.50 (11.10) | 23.30 (3.40) | 54.52 (12.76) | 114.46 (30.94) | 220.42 (40.22) | 137.24 (158.49) |
| CCHS | 14,739 | 4,222/10,517 | 52.49 (8.70) | 24.77 (3.69) | 51.20 (12.42) | 103.82 (29.73) | 183.17 (34.93) | 149.20 (110.61) |
| **Summary** | **23,083** | **9,447/13,636** | **46.46 (9.31)** | **24.25 (3.48)** | **51.22 (12.26)** | **104.67 (30.25)** | **186.09 (36.32)** | **147.38 (112.33)** |

CAS: The China Atherosclerosis Study, BAS: The Beijing Atherosclerosis Study, GenSalt: The Genetic Epidemiology Network of Salt-Sensitivity study, FAMHES: The Guangxi Fangchenggang Area Male Health and Examination Survey, CCHS: The China Cardiovascular Health Study; BMI: body mass index, HDL: high density lipoprotein, LDL: low density lipoprotein, TC: total cholesterol, TG: triglycerides. The value in parentheses is standard deviation.

**Table S12.** Index SNPs used as instruments for HDL, LDL, TC and TG and their effects, standard errors for ASL for the Chinese population

| SNP | CHR | Position (GRCh37) | Lipid | | | | | |  | ALS | | |
| --- | --- | --- | --- | --- | --- | --- | --- | --- | --- | --- | --- | --- |
|  |  |  | Allele | Beta | SE | *p* | PVE | *F* |  | Allele | Beta | SE |
| **HDL** |  |  |  |  |  |  |  |  |  |  |  |  |
| rs12678919 | 8 | 19,986,711 | A | -0.1594 | 0.0167 | 1.02E-21 | 3.93E-3 | 91.10 |  | A | -0.0116 | 0.0187 |
| rs2575876 | 9 | 104,903,458 | G | 0.0821 | 0.0115 | 7.50E-13 | 2.20E-3 | 50.96 |  | G | 0.0116 | 0.0126 |
| rs662799 | 11 | 116,792,991 | G | -0.2053 | 0.0102 | 4.20E-90 | 1.72E-2 | 405.08 |  | G | 0.0039 | 0.0117 |
| rs1800588 | 15 | 58,431,476 | T | 0.1193 | 0.0096 | 1.25E-35 | 6.65E-3 | 154.42 |  | T | -0.0023 | 0.0105 |
| rs3764261 | 16 | 56,959,412 | A | 0.2735 | 0.0129 | 9.63E-100 | 1.91E-2 | 449.47 |  | A | -0.0003 | 0.0138 |
| rs12970066 | 18 | 49,580,782 | G | 0.0640 | 0.0104 | 7.92E-10 | 1.64E-3 | 37.87 |  | G | -0.0061 | 0.0114 |
| **LDL** |  |  |  |  |  |  |  |  |  |  |  |  |
| rs7525649 | 1 | 55,033,483 | T | 0.0646 | 0.0095 | 1.21E-11 | 2.00E-3 | 46.24 |  | T | -0.0063 | 0.0107 |
| rs312949 | 2 | 21,111,411 | C | 0.0468 | 0.0100 | 3.14E-6 | 9.48E-4 | 21.90 |  | C | 0.0002 | 0.0114 |
| rs6871667 | 5 | 75,308,917 | A | 0.0732 | 0.0107 | 4.39E-13 | 2.02E-3 | 46.80 |  | A | -0.0178 | 0.0100 |
| rs9357121 | 6 | 31,272,702 | T | 0.0609 | 0.0100 | 9.41E-10 | 1.60E-3 | 37.08 |  | T | -0.0075 | 0.0140 |
| rs579459 | 9 | 133,278,724 | C | 0.0943 | 0.0130 | 3.81E-13 | 2.27E-3 | 52.61 |  | C | 0.0095 | 0.0123 |
| rs12927205 | 16 | 71,991,178 | A | 0.0483 | 0.0102 | 2.18E-6 | 9.70E-4 | 22.42 |  | A | -0.0345 | 0.0114 |
| rs7258950 | 19 | 11,139,463 | G | 0.1120 | 0.0108 | 3.30E-25 | 4.64E-3 | 107.54 |  | G | -0.0085 | 0.0124 |
| **TC** |  |  |  |  |  |  |  |  |  |  |  |  |
| rs7525649 | 1 | 55,033,483 | T | 0.0609 | 0.0093 | 7.05E-11 | 1.85E-3 | 42.88 |  | T | -0.0063 | 0.0107 |
| rs12042319 | 1 | 62,584,148 | G | 0.0614 | 0.0111 | 3.20E-8 | 1.32E-3 | 30.60 |  | G | 0.0181 | 0.0127 |
| rs312949 | 2 | 21,111,411 | C | 0.0439 | 0.0098 | 6.95E-6 | 8.69E-4 | 20.07 |  | C | 0.0002 | 0.0114 |
| rs1260333 | 2 | 27,525,757 | A | 0.0488 | 0.0088 | 2.70E-8 | 1.33E-3 | 30.75 |  | A | -0.0016 | 0.0101 |
| rs6871667 | 5 | 75,308,917 | A | 0.0609 | 0.0089 | 8.27E-12 | 2.02E-3 | 46.82 |  | A | -0.0178 | 0.0100 |
| rs9357121 | 6 | 31,272,702 | T | 0.0568 | 0.0091 | 3.71E-10 | 1.68E-3 | 38.96 |  | T | -0.0075 | 0.0140 |
| rs2954029 | 8 | 125,478,730 | A | 0.0611 | 0.0089 | 7.59E-12 | 2.04E-3 | 47.13 |  | A | -0.0091 | 0.0102 |
| rs2575876 | 9 | 104,903,458 | G | 0.0888 | 0.0107 | 1.45E-16 | 2.97E-3 | 68.87 |  | G | 0.0116 | 0.0126 |
| rs579459 | 9 | 133,278,724 | C | 0.0836 | 0.0132 | 2.38E-10 | 1.73E-3 | 40.11 |  | C | 0.0095 | 0.0123 |
| rs1800588 | 15 | 58,431,476 | T | 0.0749 | 0.0091 | 2.43E-16 | 2.93E-3 | 67.74 |  | T | -0.0023 | 0.0105 |
| rs3764261 | 16 | 56,959,412 | A | 0.1222 | 0.0124 | 4.75E-23 | 4.19E-3 | 97.11 |  | A | -0.0003 | 0.0138 |
| rs12927205 | 16 | 71,991,178 | A | 0.0491 | 0.0100 | 8.44E-7 | 1.04E-3 | 24.11 |  | A | -0.0345 | 0.0114 |
| rs12453914 | 17 | 69,142,737 | A | 0.0341 | 0.0077 | 8.64E-6 | 8.49E-4 | 19.61 |  | A | 0.0045 | 0.0103 |
| rs7258950 | 19 | 11,139,463 | G | 0.0872 | 0.0105 | 1.24E-16 | 2.98E-3 | 68.96 |  | G | -0.0085 | 0.0124 |
| **TG** |  |  |  |  |  |  |  |  |  |  |  |  |
| rs12042319 | 1 | 62,584,148 | G | 0.0200 | 0.0031 | 6.43E-11 | 1.80E-3 | 41.62 |  | G | 0.0181 | 0.0127 |
| rs1260333 | 2 | 27,525,757 | A | 0.0220 | 0.0020 | 4.28E-27 | 5.21E-3 | 120.99 |  | A | -0.0016 | 0.0101 |
| rs13231516 | 7 | 73,448,919 | T | 0.0300 | 0.0041 | 1.98E-13 | 2.31E-3 | 53.53 |  | T | 0.0284 | 0.0154 |
| rs12678919 | 8 | 19,986,711 | A | 0.0440 | 0.0041 | 4.28E-27 | 4.96E-3 | 115.16 |  | A | -0.0116 | 0.0187 |
| rs2954029 | 8 | 125,478,730 | A | 0.0230 | 0.0020 | 1.85E-29 | 5.70E-3 | 132.24 |  | A | -0.0091 | 0.0102 |
| rs662799 | 11 | 116,792,991 | G | 0.0810 | 0.0031 | 2.80E-154 | 2.87E-2 | 682.67 |  | G | 0.0039 | 0.0117 |
| rs11066280 | 12 | 112,379,979 | T | 0.0170 | 0.0031 | 2.80E-8 | 1.30E-3 | 30.07 |  | T | -0.0078 | 0.0106 |
| rs1800588 | 15 | 58,431,476 | T | 0.0180 | 0.0020 | 1.14E-18 | 3.50E-3 | 80.99 |  | T | -0.0023 | 0.0105 |
| rs157582 | 19 | 44,892,962 | T | 0.0780 | 0.0082 | 1.24E-21 | 3.90E-3 | 90.47 |  | T | -0.0034 | 0.0130 |

In the Allele column, only the effect allele is presented. TG: triglycerides, ALS: amyotrophic lateral sclerosis, Chr: chromosome, SNP: single-nucleotide polymorphism, Beta: SNP effect size, SE: standard error. The estimated proportion of variance explained (PVE) by the selected SNPs are 5.08%, 1.45%, 2.78% and 5.74% for HDL, LDL, TC and TG, respectively. We compute the *F* statistic for each instrument via $F_{i} = \text{PVE}_{i}\text{(}N_{i} -1-k\text{)/}(k-k\times\text{PVE}_{i})$, where *N_i_* is the sample size for instrument *i* and *k* is the number of total instruments. All the *F* statistics are larger than 10 (ranging from 19.61 to 682.67). The overall *F* statistics are 205.83, 48.50, 47.12 and 156.12 for HDL, LDL, TC and TG, respectively.

**Table S13.** The 63 studies used in the global lipids genetics consortium of European population

| Short study name | *N* | Female (%) | Age | HDL (mg/dL) | LDL (mg/dL) | TC (mg/dL) | TG (mg/dL) |
| --- | --- | --- | --- | --- | --- | --- | --- |
| ADVANCE | 505 | 40.6 | 65.7 (2.9) | 54.6 (16.5) | 128.3 (29.9) | 209.7 (34.5) | 137.7 (87.6) |
| AIDHS/SDS | 1,516 | 47.2 | 53.0 (12.1) | 38.8 (13.8) | 108.9 (38.7) | 181.5 (49.2) | 181.5 (116.7) |
| AMC-PAS | 304 | 25.0 | 43.0 (5.4) | 44.2 (14.8) | 148.9 (46.0) | 234.2 (59.9) | 167.1 (112.9) |
| AMISH | 1,081 | 50.0 | 46.7 (15.1) | 54.2 (11.6) | 135.5 (38.7) | 209.0 (42.6) | 74.4 (44.3) |
| BC58 | 2,136 | 57.8 | 45.0 (0.0) | 60.0 (15.1) | 134.7 (35.6) | 230.3 (41.8) | 185.2 (130.2) |
| CLHNS^#^ | 1,771 | 47.3 | 21.5 (0.3) | 42.3 (11.2) | 94.0 (29.2) | 157.2 (36.6) | 105.5 (65.6) |
| D2D 2007 (T2D) | 287 | 43.6 | 62.3 (7.8) | 50.8 (12.4) | 132.4 (34.5) | 213.2 (41.2) | 151.1 (90.8) |
| D2D 2007 (controls) | 1,821 | 56.3 | 58.3 (8.3) | 57.4 (13.4) | 138.0 (31.3) | 218.0 (35.9) | 114.6 (70.6) |
| deCODE | 15,612 | 62.8 | 60.9 (17.1) | 56.0 (17.9) | 135.2 (39.9) | 217.3 (43.8) | 134.7 (81.8) |
| DIAGEN (T2D) | 439 | 50.3 | 66.0 (11.9) | 47.5 (16) | 114.0 (38.4) | 198.7 (48.2) | 199.6 (176.4) |
| DIAGEN (controls) | 1,093 | 56.7 | 62.4 (15.2) | 59.3 (17.9) | 127.7 (38.4) | 207.2 (46.4) | 135.0 (170.3) |
| DILGOM | 3,738 | 58.1 | 51.6 (13.5) | 56.1 (13.5) | 122.3 (32.1) | 202.0 (36.8) | 104.5 (62.9) |
| DPS (T2D) | 85 | 63.5 | 55.1 (6.4) | 44.0 (11.7) | 134.4 (32.8) | 212.2 (37.7) | 172.3 (85.5) |
| DPS (controls) | 362 | 69.6 | 55.2 (7.3) | 47.9 (11.1) | 141.5 (31.2) | 218.3 (34.2) | 147.2 (61.8) |
| DRAGON (TAICHI)^#^ | 1,052 | 41.7 | 62.9 (14.6) | 43.3 (16.3) | 101.9 (40.6) | 174.5 (47.8) | 146.6 (100.1) |
| DR'S EXTRA (T2D) | 121 | 50.4 | 68.7 (5.8) | 58.3 (17.4) | 112.1 (33.6) | 181.1 (36.4) | 142.6 (73.3) |
| DR'S EXTRA (controls) | 1,174 | 53.8 | 66.4 (5.3) | 66.8 (18.6) | 125.6 (32.2) | 198.7 (35.9) | 115.5 (58.9) |
| EAS | 733 | 53.0 | 64.4 (5.7) | 55.7 (13.2) | 206.7 (47.2) | 274.4 (51.1) | 461.6 (152.4) |
| EGCUT | 1,240 | 53.5 | 64.2 (10.9) | 53.8 (27.9) | 136.6 (44.1) | 211.3 (46.8) | 161.3 (111.6) |
| Ely | 1,602 | 53.6 | 61.1 (9.2) | 56.5 (15.5) | 135.5 (36.8) | 215.6 (41.0) | 128.5 (74.4) |
| EPIC-CAD cases (EPIC-Norfolk CAD set) | 1,529 | 35.1 | 65.2 (7.9) | 49.9 (14.3) | 164.1 (40.2) | 250.8 (46.4) | 192.3 (108.1) |
| EPIC-T2D cases (EPIC-Norfolk T2D set) | 700 | 40.3 | 62.2 (8.3) | 46.4 (12.8) | 153.3 (40.2) | 245.4 (48.4) | 243.7 (140.9) |
| EPIC-T2D controls | 994 | 56.8 | 59.4 (9.4) | 55.0 (16.3) | 154.0 (38.3) | 239.2 (43.0) | 156.8 (89.5) |
| FBPP | 1,614 | 64.0 | 43.9 (12.5) | 53.6 (15.8) | 118.4 (37.8) | 193.2 (43.5) | NA |
| Fenland | 3,186 | 53.3 | 46.9 (7.1) | 58.8 (15.1) | 129.3 (34.8) | 207.0 (39.5) | 103.7 (71.8) |
| FINCAVAS | 1,201 | 44.4 | 58.5 (12.6) | 54.6 (17.8) | 113.0 (33.3) | 191.2 (38.7) | 119.6 (65.6) |
| FRISCII | 2,963 | 30.6 | 66.2 (9.7) | 46.7 (15.2) | 145.7 (38.2) | 228.1 (43.1) | 185.7 (119.5) |
| FUSION2 (T2D) | 843 | 43.3 | 59.5 (8.5) | 51.1 (14.8) | 123.1 (36.2) | 217.6 (47.3) | 163.3 (92.8) |
| FUSION2 (controls) | 1,880 | 44.7 | 56.2 (8.2) | 58.1 (15.8) | 137.9 (34.9) | 225.7 (42.5) | 114.8 (68.5) |
| GLACIER | 5,764 | 61.4 | 54.2 (8.0) | 56.3 (13.4) | 173.0 (45.3) | 232.5 (47.4) | 142.6 (47.4) |
| Go-DARTs | 6,759 | 46.1 | 57.8 (10.3) | 55.0 (17.0) | 120.7 (35.2) | 216.7 (46.8) | 177.2 (126.7) |
| GXE^#^ | 397 | 76.8 | 39.8 (8.2) | 50.9 (12.0) | 146.1 (41.6) | 204.3 (43.7) | 84.7 (50.1) |
| HALST (TAICHI) ^#^ | 2,375 | 49.4 | 68.9 (8.3) | 52.6 (13.5) | 120.0 (31.8) | 197.7 (36.8) | 123.1 (76.2) |
| HUNT (T2D) | 588 | 49.5 | 69.3 (11.3) | 45.8 (14.6) | 151.9 (42.0) | 241.7 (51.8) | 238.7 (163.4) |
| HUNT (controls) | 784 | 49.0 | 66.3 (14.4) | 50.9 (14.3) | 163.4 (44.1) | 249.7 (48.3) | 183.8 (101.1) |
| IMPROVE | 1,769 | 50.0 | 64.4 (5.3) | 48.8 (14.7) | 148.6 (36.4) | 224.1 (42.2) | 139.1 (96.6) |
| KORA F3 | 2,816 | 52.1 | 56.3 (12.8) | 59.2 (18.2) | 129.6 (32.1) | 220.2 (39.5) | 163.9 (124.9) |
| KORA F4 | 2,678 | 53.1 | 54.5 (13.1) | 56.5 (14.7) | 138.5 (34.8) | 218.3 (39.5) | 122.3 (86.8) |
| MRC/UVRIGPCad^#^ | 1,687 | 56.7 | 35.0 (19.1) | 39.9 (14.3) | 78.6 (29.0) | 138.9 (36.8) | 104.5 (55.8) |
| LURIC (cases) | 983 | 26.4 | 64.9 (9.7) | 38.5 (10.7) | 124.5 (33.4) | 201.2 (37.3) | 170.0 (99.9) |
| LURIC (controls) | 523 | 46.1 | 57.9 (12.5) | 42.8 (11.5) | 121.2 (29.4) | 198.8 (35.0) | 154.5 (100.9) |
| MDC | 2,125 | 51.3 | 57.4 (6.0) | 53.9 (13.9) | 160.0 (36.6) | 235.5 (40.2) | 109.2 (50.5) |
| METSIM (T2D) | 634 | 0.0 | 59.7 (6.8) | 52.1 (16.1) | 134.6 (36.2) | 212.8 (44.4) | 167.0 (111.8) |
| METSIM (controls) | 829 | 0.0 | 53.7 (5.0) | 57.3 (14.7) | 138.4 (29.9) | 215.3 (34.0) | 120.1 (76.2) |
| NFBC86 | 4,164 | 52 | 16.0 (0.4) | 54.6 (11.2) | 87.1 (22.4) | 164.9 (30.2) | 73.5 (36.3) |
| NSHD | 941 | 52.8 | 53.0 (63.5) | 19.7 (136.2) | 38.3 (237.2) | 43.0 (206.4) | 154.2 (NA) |
| PIVUS | 854 | 51.0 | 70.0 (0.2) | 58.1 (15.5) | 135.5 (31.0) | 216.7 (38.7) | 106.3 (44.3) |
| PROMIS^#^ | 3,385 | 18.0 | 52.5 (9.9) | 35.5 (9.9) | 122.3 (43.7) | 192.7 (50.7) | 210.2 (128.8) |
| SAPPHIRe (TAICHI) ^#^ | 251 | 49.4 | 54.6 (10.5) | 44.2 (12.6) | 127.1 (38.2) | 200.4 (43.4) | 143.0 (80.0) |
| SardiNIA | 5,378 | 56.8 | 43.2 (17.4) | 64.3 (14.9) | 127.1 (35.5) | 208.4 (42.6) | 86.9 (68.3) |
| SCARFSHEEP | 2,973 | 0.3 | 58.3 (7.2) | 45.7 (13.9) | 156.3 (37.5) | 230.7 (42.2) | 148.0 (101.0) |
| SEY | 426 | 54.7 | 48.7 (14.1) | 48.3 (13.1) | 141.4 (44.1) | 213.3 (49.1) | 117.8 (80.1) |
| SPT | 838 | 61.8 | 46.7 (0.5) | 48.8 (13.0) | 133.7 (41.3) | 192.8 (42.1) | 89.1 (53.8) |
| STR | 2,543 | 57.0 | 75.0 (10.2) | 54.2 (15.5) | 150.9 (42.6) | 243.8 (50.3) | 150.6 (79.7) |
| TACT (TAICHI) ^#^ | 173 | 31.2 | 64.1 (10.7) | 34.3 (8.0) | 103.5 (24.3) | 180.1 (36.8) | 130.0 (74.2) |
| TCAD (TAICHI) ^#^ | 2,284 | 23.3 | 65.8 (11.7) | 44.0 (12.5) | 112.3 (40.7) | 182.6 (42.4) | 133.2 (84.7) |
| TCAGEN (TAICHI) ^#^ | 383 | 34.7 | 64.3 (13.3) | 45.5 (17.7) | 112.1 (37.0) | 185.5 (44.6) | 166.9 (131.6) |
| THISEAS | 929 | 50.7 | 58.6 (13.5) | 52.7 (15.7) | 134.5 (38.1) | 211.9 (42.6) | 127.1 (77.9) |
| TROMSO (T2D) | 710 | 50.4 | 60.0 (12.5) | 51.2 (14.8) | 168.4 (42.2) | 260.5 (46.9) | 223.3 (141.5) |
| TROMSO (controls) | 711 | 50.2 | 60.0 (12.5) | 59.5 (16.4) | 166.9 (43.7) | 254.9 (48.3) | 145.3 (89.9) |
| TUDR (TAICHI)^#^ | 669 | 45.7 | 64.6 (12.1) | 42.0 (15.0) | 105.5 (40.5) | 178.2 (52.7) | 151.5 (97.6) |
| ULSAM | 1,113 | 0.0 | 71.0 (0.6) | 50.3 (11.6) | 150.9 (34.8) | 224.5 (38.7) | 124.0 (70.9) |
| WHII | 3,212 | 23.0 | 48.9 (6.0) | 53.4 (28.3) | 160.2 (78.6) | 249.2 (43.3) | 129.4 (101.9) |
| **Summary** | **114,230** | **50.2** | **58.6 (9.7)** | **52.1 (14.7)** | **134.5 (37.0)** | **212.8 (42.6)** | **142.8 (86.8)** |

HDL: high density lipoprotein, LDL: low density lipoprotein, TC: total cholesterol, TG: triglycerides. The value in parentheses is standard deviation. The information is obtained from . Due to the qulity control of the data set, the summation of the sample sizes of these 63 studies (i.e. 114,230) is less than the effective sample size 93,982 in the main text; the remaining 94,595 individuals from previous 23 studies , resulting in the total sample size up to ~190,000. # denotes studyeis with non-European ancestry.

**Table S14.** The 49 studies used in the GWAS of amyotrophic lateral sclerosis for the European population

| Studies | Case/Control |
| --- | --- |
| **GWAS discovery phase sample ascertainment**  NL1: Population based ALS registry, The Netherlands | 461/450 |
| BE1: University Hospital Gasthuisberg Leuven, Belgium | 311/371 |
| NL2: Population based ALS registry, The Netherlands | 582/629 |
| SW1: Umeå University ALS clinic, Sweden | 493/500 |
| NL3: Rotterdam Study, Erasmus University Medical Centre Rotterdam, The Netherlands | 0/5,974 |
| FR1: Hôpitaux de Paris, Hôpital de la Salpêtrière Paris, France | 251/724 |
| UK1: Institute of Psychiatry, King’s College London, United Kingdom | 245/221 |
| US1: Massachusetts General Hospital Boston and Emory University Hospital Atlanta, | 753/811 |
| IR1: Population based ALS registry, Ireland | 221/211 |
| IR2: Population based ALS registry, Ireland | 103/127 |
| UK2: UK Biobank for Motor Neuron Disease Research, United Kingdom | 661/0 |
| US2: Coriell Institute for Medical Research, United States of America | 0/527 |
| US3: Coriell Institute for Medical Research, United States of America | 276/0 |
| IT1: Population based registry (PARALS), Italy | 141/0 |
| IT2: Coriell Institute for Medical Research, United States of America | 261/246 |
| UK3: Wellcome Trust Case Control Consortium UK National Blood Service, United | 0/2,501 |
| UK4: Wellcome Trust Case Control Consortium 1958 Birth Cohort, United Kingdom | 0/2,699 |
| US4: Coriell Institute for Medical Research, United States of America | 0/867 |
| US5: The NeuroGenetics Research Consortium (NGRC), United States of America | 0/1,986 |
| FIN1: Population-based cohort, Finland | 0/201 |
| FIN2: Helsinki University Central Hospital, Finland | 401/191 |
| FIN3: Population-based cohort, Finland | 0/103 |
| IT3: Italian Consortium for the Genetics of ALS (SLAGEN), Italy | 1,792/1,107 |
| FR2: 3C study, Population-based French controls, France | 0/1,100 |
| GER1: PopGen, Population-based German controls, Germany | 0/677 |
| NL4 Population based ALS registry, The Netherlands | 1,226/2,262 |
| GER2: Hannover Medical School and University of Wuerzburg, Germany | 580/286 |
| IT4: Population based ALS registries (SLALOM and SLAP), Italy | 311/100 |
| PU1: University of Lisbon, Portugal | 40/54 |
| SP1: University of Madrid, University of Barcelona, Spain | 105/63 |
| SWISS1: Kantonsspital St Gallen, Switzerland | 228/236 |
| BE2: University Hospital Gasthuisberg Leuven, Belgium | 225/250 |
| FIN4: ALS clinics, Finland (via Umeå University, Sweden) | 144/0 |
| IR3: Population based ALS registry, Ireland | 268/478 |
| SW2: ALS clinics, via Umeå University, Sweden | 281/271 |
| US6: University of California Los Angeles, United States of America | 65/42 |
| GER3: University of Ulm, Germany | 1,519/0 |
| FR3: Paris and Tours, France | 363/0 |
| US7: Massachusetts General Hospital, Boston, Emory University,  Atlanta and the Penn ALS Center, University of Pennsylvania, United States of America | 573/0 |
| UK5: UK Biobank for Motor Neuron Disease Research, United Kingdom | 1,214/14 |
| NL5: Population based ALS registry, The Netherlands | 697/619 |
| **SUMMARY** | **14,791/26,898** |
|  |  |
| **GWAS replication phase sample ascertainment** |  |
| Sydney Australia | NA |
| University Hospital Gasthuisberg Leuven, Belgium | NA |
| University Hospital Tours, France | NA |
| Jena Germany | NA |
| Population based ALS registry, Ireland | NA |
| Population based ALS registries, Italy | NA |
| Population based ALS registry, The Netherlands | NA |
| Population based ALS registry, Turkey | NA |
| **Summary** | **2,579/2,767** |

In the GWAS discovery phase, the total sample sizes for cases and controls are slightly larger than those reported in the original paper (i.e. 14,791/26,898 vs. 12,577/23,475) due to quality control for the data.

**Table S15.** Baseline Characteristics of the East Asian lipids GWAS

| Trait | HDL | LDL | TC | TG |
| --- | --- | --- | --- | --- |
| Sample Size | 70,657 | 72,866 | 128,305 | 105,597 |
| Male/Female | 40,557/30,100 | 41,679/31,187 | 70,568/57,737 | 59,029/46,568 |
| Mean Age | 63.7 (12.1) | 63.9 (12.0) | 63.1 (12.9) | 63.6 (12.5) |
| Mean of the trait (mg/dL) | 54.75 (15.43) | 130.81 (40.97) | 199.17 (38.56) | 133.17 (71.86) |

HDL: high density lipoprotein, LDL: low density lipoprotein, TC: total cholesterol, TG: triglycerides. The value in parentheses is standard deviation.

**Figure S1.** Graphical illustration of Mendelian randomization analysis illustrates three key modeling assumptions. Solid arrows or dotted arrows denote the presence or absence of directional associations. The Mendelian randomization analysis estimates the causal effect of each lipid trait to ALS in the presence of various measured and unmeasured confounding factors by carefully selecting SNPs that are associated with the lipid trait to serve as instrumental variables. Valid Mendelian randomizations require these selected SNPs to satisfy three modeling assumptions. **Assumption 1**, known as the relevance condition, assumes that the selected SNPs are strongly associated with the outcome (lipid trait). **Assumption 2**, known as the independence condition, assumes that the selected SNPs are not associated with the any known or known confounders of the lipid trait and ALS. **Assumption 3**, known as the exclusion restriction, assumes that the selected SNPs are independent of ALS conditional on the lipid. SNP: single nucleotide polymorphisms; HDL: high density lipoprotein; LDL: low density lipoprotein; TC: total cholesterol; TG: triglycerides; ALS: amyotrophic lateral sclerosis.

(**A**)	(**B**)

(**C**)	(**D**)

**Figure S2.** Venn diagram displays the pleiotropic association pattern of the selected instruments or top associated SNPs for the four lipids. (**A**) The pleiotropic association pattern of the selected instruments for the four lipids in the European population. For example, for the selected 78 LDL associated instruments, 39 are associated with TC, 3 are associated with both TC and HDL, 1 is associated with TC and TG, while 1 is associated with both HDL and TG. (**B**) The pleiotropic association pattern of all genome-wide significant SNPs listed in for the four lipids in the European population. (**C**) The pleiotropic association pattern of the selected instruments for the four lipids in the East Asian population. (**D**) The pleiotropic association pattern of all genome-wide significant SNPs listed in for the four lipids in the East Asian population.

**Figure S3.** Estimated causal effects of LDL on ALS in the leave-one-out analysis using the fixed-effects IVW method in the European population (**A**) and the East Asian population (**B**). For example, in the European population, after removing rs11591147, which has the largest effect on LDL among all instruments (effect size = 0.50, 95% CI 0.46 - 0.53, *p* = 8.58E-143), the OR of ALS per unit increase of LDL is still estimated to be 1.14 (95% CI 1.06 - 1.26, *p* = 3.00E-4) by the fixed-effects IVW analysis, which is almost identical to the estimate obtained using all instruments

**Figure S4.** Causal effect estimates and 95% confidence intervals for four lipids on ALS for the Chinese population. In the plot, center of the dot represents estimated causal effects and the magnitude of the dot is in proportion to the number of the instrumental variables used in the analysis. Causal effects with a *p* value below a nominal level of 0.05 are highlighted in red. Name of the traits that have a causal effect *p* value below a Bonferroni corrected threshold (*p* < 0.05/4) are further colored in red on the x-axis. For each lipid, the left dot shows results using the fixed-effects IVW method and the right dot shows causal results using the random-effects IVW method.

## References

1.	Sutedja, N.A., van der Schouw, Y.T., Fischer, K., Sizoo, E.M., Huisman, M.H.B., Veldink, J.H. and Van den Berg, L.H. (2011) Beneficial vascular risk profile is associated with amyotrophic lateral sclerosis. *J Neurol Neurosurg Psychiatry*, **82**, 638-642.

2.	O'Reilly, É.J., Wang, H., Weisskopf, M.G., Fitzgerald, K.C., Falcone, G., McCullough, M.L., Thun, M., Park, Y., Kolonel, L.N. and Ascherio, A. (2013) Premorbid body mass index and risk of amyotrophic lateral sclerosis. *Amyotrophic Lateral Sclerosis and Frontotemporal Degeneration*, **14**, 205-211.

3.	Gallo, V., Wark, P.A., Jenab, M., Pearce, N., Brayne, C., Vermeulen, R., Andersen, P.M., Hallmans, G., Kyrozis, A., Vanacore, N. *et al.* (2013) Prediagnostic body fat and risk of death from amyotrophic lateral sclerosis The EPIC cohort. *Neurology*, **80**, 829-838.

4.	Gianfrancesco, M.A., Glymour, M.M., Walter, S., Rhead, B., Shao, X., Shen, L., Quach, H., Hubbard, A., Jónsdóttir, I., Stefánsson, K. *et al.* (2017) Causal Effect of Genetic Variants Associated With Body Mass Index on Multiple Sclerosis Susceptibility. *Am J Epidemiol*, **185**, 162-171.

5.	Holmes, Michael V., Lange, Leslie A., Palmer, T., Lanktree, Matthew B., North, Kari E., Almoguera, B., Buxbaum, S., Chandrupatla, Hareesh R., Elbers, Clara C., Guo, Y. *et al.* (2014) Causal Effects of Body Mass Index on Cardiometabolic Traits and Events: A Mendelian Randomization Analysis. *Am J Hum Genet*, **94**, 198-208.

6.	Panoutsopoulou, K. (2014) The effect of FTO variation on increased osteoarthritis risk is mediated through body mass index: a mendelian randomisation study. *Ann Rheum Dis*, **73**, 2082-2086.

7.	Dixon, S.C., Nagle, C.M., Thrift, A.P., Pharoah, P.D., Pearce, C.L., Zheng, W., Painter, J.N., Group, A., Study, A.C., Chenevix-Trench, G. *et al.* (2016) Adult body mass index and risk of ovarian cancer by subtype: a Mendelian randomization study. *Int J Epidemiol*, **45**, 884-895.

8.	Tsilidis, K.K., Dai, J.Y. and Peters, U. (2017) Mendelian Randomization Analysis Identifies Body Mass Index and Fasting Insulin as Potential Causal Risk Factors for Pancreatic Cancer Risk. *JNCI: Journal of the National Cancer Institute*, in press.

9.	Guo, Y., Warren Andersen, S., Shu, X.-O., Michailidou, K., Bolla, M.K., Wang, Q., Garcia-Closas, M., Milne, R.L., Schmidt, M.K., Chang-Claude, J. *et al.* (2016) Genetically Predicted Body Mass Index and Breast Cancer Risk: Mendelian Randomization Analyses of Data from 145,000 Women of European Descent. *PLoS Med*, **13**, e1002105.

10.	Mendelson, M.M., Marioni, R.E., Joehanes, R., Liu, C., Hedman, Å.K., Aslibekyan, S., Demerath, E.W., Guan, W., Zhi, D., Yao, C. *et al.* (2017) Association of Body Mass Index with DNA Methylation and Gene Expression in Blood Cells and Relations to Cardiometabolic Disease: A Mendelian Randomization Approach. *PLoS Med*, **14**, e1002215.

11.	Noyce, A.J., Kia, D.A., Hemani, G., Nicolas, A., Price, T.R., De Pablo-Fernandez, E., Haycock, P.C., Lewis, P.A., Foltynie, T., Davey Smith, G. *et al.* (2017) Estimating the causal influence of body mass index on risk of Parkinson disease: A Mendelian randomisation study. *PLoS Med*, **14**, e1002314.

12.	Lawlor, D.A., Harbord, R.M., Sterne, J.A., Timpson, N. and Davey Smith, G. (2008) Mendelian randomization: using genes as instruments for making causal inferences in epidemiology. *Stat Med*, **27**, 1133-1163.

13.	Sheehan, N.A., Didelez, V., Burton, P.R. and Tobin, M.D. (2008) Mendelian randomisation and causal inference in observational epidemiology. *PLoS Med*, **5**, e177.

14.	Visscher, P.M., Wray, N.R., Zhang, Q., Sklar, P., McCarthy, M.I., Brown, M.A. and Yang, J. (2017) 10 Years of GWAS Discovery: Biology, Function, and Translation. *Am J Hum Genet*, **101**, 5-22.

15.	Welter, D., MacArthur, J., Morales, J., Burdett, T., Hall, P., Junkins, H., Klemm, A., Flicek, P., Manolio, T., Hindorff, L. *et al.* (2014) The NHGRI GWAS Catalog, a curated resource of SNP-trait associations. *Nucleic Acids Res*, **42**, D1001-D1006.

16.	Teslovich, T.M., Musunuru, K., Smith, A.V., Edmondson, A.C., Stylianou, I.M., Koseki, M., Pirruccello, J.P., Ripatti, S., Chasman, D.I., Willer, C.J. *et al.* (2010) Biological, clinical and population relevance of 95 loci for blood lipids. *Nature*, **466**, 707-713.

17.	Willer, C.J., Schmidt, E.M., Sengupta, S., Peloso, G.M., Gustafsson, S., Kanoni, S., Ganna, A., Chen, J., Buchkovich, M.L., Mora, S. *et al.* (2013) Discovery and refinement of loci associated with lipid levels. *Nat Genet*, **45**, 1274–1283.

18.	Paternoster, L., Tilling, K. and Davey Smith, G. (2017) Genetic epidemiology and Mendelian randomization for informing disease therapeutics: Conceptual and methodological challenges. *PLoS Genet*, **13**, e1006944.

19.	Sleiman, P.M. and Grant, S.F. (2010) Mendelian randomization in the era of genomewide association studies. *Clin Chem*, **56**, 723-728.

20.	Purcell, S., Neale, B., Todd-Brown, K., Thomas, L., Ferreira, M.A.R., Bender, D., Maller, J., Sklar, P., de Bakker, P.I.W., Daly, M.J. *et al.* (2007) PLINK: A Tool Set for Whole-Genome Association and Population-Based Linkage Analyses. *Am J Hum Genet*, **81**, 559-575.

21.	The 1000 Genomes Project Consortium (2015) A global reference for human genetic variation. *Nature*, **526**, 68-74.

22.	van Rheenen, W., Shatunov, A., Dekker, A.M., McLaughlin, R.L., Diekstra, F.P., Pulit, S.L., van der Spek, R.A., Võsa, U., de Jong, S. and Robinson, M.R. (2016) Genome-wide association analyses identify new risk variants and the genetic architecture of amyotrophic lateral sclerosis. *Nat Genet*, **48**, 1043-1048.

23.	Kanai, M., Akiyama, M., Takahashi, A., Matoba, N., Momozawa, Y., Ikeda, M., Iwata, N., Ikegawa, S., Hirata, M., Matsuda, K. *et al.* (2018) Genetic analysis of quantitative traits in the Japanese population links cell types to complex human diseases. *Nat Genet*, **50**, 390-400.

24.	Benyamin, B., He, J., Zhao, Q., Gratten, J., Garton, F., Leo, P.J., Liu, Z., Mangelsdorf, M., Al-Chalabi, A. and Anderson, L. (2017) Cross-ethnic meta-analysis identifies association of the GPX3-TNIP1 locus with amyotrophic lateral sclerosis. *Nat Commun*, **8**, 611.

25.	Yavorska, O.O. and Burgess, S. (2017) MendelianRandomization: an R package for performing Mendelian randomization analyses using summarized data. *Int J Epidemiol*, in press., dyx034.

26.	Bowden, J., Del Greco M, F., Minelli, C., Davey Smith, G., Sheehan, N.A. and Thompson, J.R. (2016) Assessing the suitability of summary data for two-sample Mendelian randomization analyses using MR-Egger regression: the role of the I^2^ statistic. *Int J Epidemiol*, **45**, 1961-1974.

27.	Burgess, S., Small, D.S. and Thompson, S.G. (2017) A review of instrumental variable estimators for Mendelian randomization. *Stat Methods Med Res*, **26**, 2333-2355.

28.	Hartwig, F.P., Davey Smith, G. and Bowden, J. (2017) Robust inference in summary data Mendelian randomization via the zero modal pleiotropy assumption. *Int J Epidemiol*, **46**, 1985-1998.

29.	Brockwell, S.E. and Gordon, I.R. (2001) A comparison of statistical methods for meta-analysis. *Stat Med*, **20**, 825-840.

30.	DerSimonian, R. and Laird, N. (1986) Meta-analysis in clinical trials. *Control Clin Trials*, **7**, 177-188.

31.	Thompson, S.G. and Sharp, S.J. (1999) Explaining heterogeneity in meta-analysis: A comparison of methods. *Stat Med*, **18**, 2693-2708.

32.	Burgess, S. and Thompson, S.G. (2017) Interpreting findings from Mendelian randomization using the MR-Egger method. *Eur J Epidemiol*, **32**, 377-389.

33.	Bowden, J., Davey Smith, G., Haycock, P.C. and Burgess, S. (2016) Consistent estimation in Mendelian randomization with some invalid instruments using a weighted median estimator. *Genet Epidemiol*, **40**, 304-314.

34.	Dupuis, L., Corcia, P., Fergani, A., Gonzalez De Aguilar, J.-L., Bonnefont-Rousselot, D., Bittar, R., Seilhean, D., Hauw, J.-J., Lacomblez, L., Loeffler, J.-P. *et al.* (2008) Dyslipidemia is a protective factor in amyotrophic lateral sclerosis. *Neurology*, **70**, 1004-1009.

35.	Chio, A., Calvo, A., Ilardi, A., Cavallo, E., Moglia, C., Mutani, R., Palmo, A., Galletti, R., Marinou, K., Papetti, L. *et al.* (2009) Lower serum lipid levels are related to respiratory impairment in patients with ALS. *Neurology*, **73**, 1681-1685.

36.	Burgess, S. and Thompson, S.G. (2015) Multivariable Mendelian Randomization: The Use of Pleiotropic Genetic Variants to Estimate Causal Effects. *Am J Epidemiol*, **181**, 251-260.

37.	Burgess, S., Thompson, D.J., Rees, J.M.B., Day, F.R., Perry, J.R. and Ong, K.K. (2017) Dissecting Causal Pathways Using Mendelian Randomization with Summarized Genetic Data: Application to Age at Menarche and Risk of Breast Cancer. *Genetics*, **207**, 481-487.

38.	Do, R., Willer, C.J., Schmidt, E.M., Sengupta, S., Gao, C., Peloso, G.M., Gustafsson, S., Kanoni, S., Ganna, A. and Chen, J. (2013) Common variants associated with plasma triglycerides and risk for coronary artery disease. *Nat Genet*, **45**, 1345-1352.

39.	Martin, S.S., Blaha, M.J., Elshazly, M.B., Toth, P.P., Kwiterovich, P.O., Blumenthal, R.S. and Jones, S.R. (2013) Comparison of a Novel Method vs the Friedewald Equation for Estimating Low-Density Lipoprotein Cholesterol Levels From the Standard Lipid Profile. *JAMA*, **310**, 2061-2068.

40.	Lu, X., Huang, J., Mo, Z., He, J., Wang, L., Yang, X., Tan, A., Chen, S., Chen, J., Gu, C.C. *et al.* (2016) Genetic Susceptibility to Lipid Levels and Lipid Change Over Time and Risk of Incident Hyperlipidemia in Chinese Populations. *Circulation Cardiovascular Genetics*, **9**, 37-44.

41.	Scarmeas, N., Shih, T., Stern, Y., Ottman, R. and Rowland, L.P. (2002) Premorbid weight, body mass, and varsity athletics in ALS. *Neurology*, **59**, 773-775.

42.	Huisman, M.H.B., Seelen, M., de Jong, S.W., Dorresteijn, K.R.I.S., van Doormaal, P.T.C., van der Kooi, A.J., de Visser, M., Schelhaas, H.J., van den Berg, L.H. and Veldink, J.H. (2013) Lifetime physical activity and the risk of amyotrophic lateral sclerosis. *Journal of Neurology, Neurosurgery &amp; Psychiatry*, **84**, 976-981.

43.	de Jong, S.W., Huisman, M.H.B., Sutedja, N.A., van der Kooi, A.J., de Visser, M., Schelhaas, H.J., Fischer, K., Veldink, J.H. and van den Berg, L.H. (2012) Smoking, Alcohol Consumption, and the Risk of Amyotrophic Lateral Sclerosis: A Population-based Study. *Am J Epidemiol*, **176**, 233-239.

44.	Nelson, L.M., McGuire, V., Longstreth, J.W.T. and Matkin, C. (2000) Population-Based Case-Control Study of Amyotrophic Lateral Sclerosis in Western Washington State. I. Cigarette Smoking and Alcohol Consumption. *Am J Epidemiol*, **151**, 156-163.

45.	Armon, C. (2009) Smoking may be considered an established risk factor for sporadic ALS. *Neurology*, **73**, 1693-1698.

46.	Alonso, A., Logroscino, G., Jick, S.S. and Hernan, M.A. (2010) Association of smoking with amyotrophic lateral sclerosis risk and survival in men and women: a prospective study. *BMC Neurol*, **10**.

47.	Weisskopf, M.G., McCullough, M.L., Calle, E.E., Thun, M.J., Cudkowicz, M. and Ascherio, A. (2004) Prospective study of cigarette smoking and amyotrophic lateral sclerosis. *Am J Epidemiol*, **160**, 26-33.

48.	Wang, H., O'Reilly, E.J., Weisskopf, M.G., Logroscino, G., McCullough, M.L., Thun, M.J., Schatzkin, A., Kolonel, L.N. and Ascherio, A. (2011) Smoking and Risk of Amyotrophic Lateral Sclerosis A Pooled Analysis of 5 Prospective Cohorts. *Arch Neurol*, **68**, 207-213.

49.	Horner, R.D. (2003) Occurrence of amyotrophic lateral sclerosis among Gulf War veterans. *Neurology*, **61**, 742-749.

50.	Kamel, F., Umbach, D.M., Munsat, T.L., Shefner, J.M., Hu, H. and Sandler, D.P. (2002) Lead exposure and amyotrophic lateral sclerosis. *Epidemiology*, **13**, 311-319.

51.	Huisman, M.H.B., Seelen, M., van Doormaal, P.T.C., de Jong, S.W., de Vries, J.H.M., van der Kooi, A.J., de Visser, M., Schelhaas, H.J., van den Berg, L.H. and Veldink, J.H. (2015) Effect of Presymptomatic Body Mass Index and Consumption of Fat and Alcohol on Amyotrophic Lateral Sclerosis. *JAMA Neurology*, **72**, 1155-1162.

52.	Malek, A.M., Barchowsky, A., Bowser, R., Heiman-Patterson, T., Lacomis, D., Rana, S., Youk, A., Stickler, D., Lackland, D.T. and Talbott, E.O. (2014) Environmental and Occupational Risk Factors for Amyotrophic Lateral Sclerosis: A Case-Control Study. *Neurodegenerative Diseases*, **14**, 31-38.

53.	Huang, R., Guo, X., Chen, X., Zheng, Z., Wei, Q., Cao, B., Zeng, Y. and Shang, H. (2015) The serum lipid profiles of amyotrophic lateral sclerosis patients: A study from south-west China and a meta-analysis. *Amyotrophic Lateral Sclerosis and Frontotemporal Degeneration*, **16**, 359-365.

54.	Yang, J.W., Kim, S.-M., Kim, H.-J., Kim, J.-E., Park, K.S., Kim, S.-H., Lee, K.-W. and Sung, J.-J. (2013) Hypolipidemia in Patients with Amyotrophic Lateral Sclerosis: A Possible Gender Difference? *Journal of Clinical Neurology*, **9**, 125-129.

55.	Sutedja, N.A., Veldink, J.H., Fischer, K., Kromhout, H., Heederik, D., Huisman, M.H.B., Wokke, J.H.J. and van den Berg, L.H. (2009) Exposure to chemicals and metals and risk of amyotrophic lateral sclerosis: A systematic review. *Amyotroph Lateral Scler*, **10**, 302-309.

56.	Al-Chalabi, A. and Hardiman, O. (2013) The epidemiology of ALS: a conspiracy of genes, environment and time. *Nature Reviews Neurology*, **9**, 617-628.

57.	Shim, H., Chasman, D.I., Smith, J.D., Mora, S., Ridker, P.M., Nickerson, D.A., Krauss, R.M. and Stephens, M. (2015) A Multivariate Genome-Wide Association Analysis of 10 LDL Subfractions, and Their Response to Statin Treatment, in 1868 Caucasians. *PLoS ONE*, **10**, e0120758.
